# Novel indolic AMPK modulators induce vasodilatation through activation of the AMPK–eNOS–NO pathway

**DOI:** 10.1038/s41598-022-07077-8

**Published:** 2022-03-10

**Authors:** Marta Sanz-Gómez, Elnaz Aledavood, Marina Beroiz-Salaverri, Laura Lagartera, Elena Vega-Martín, Marta Gil-Ortega, Jose Cumella, Concepción Pérez, Francisco Javier Luque, Carolina Estarellas, María S. Fernández-Alfonso, Ana Castro

**Affiliations:** 1 Instituto Pluridisciplinar and Facultad de Farmacia, Universidad Complutense de Madrid, Madrid, Spain; 2 Instituto de Química Médica, IQM-CSIC, Madrid, Spain; 3 Departamento de Nutrición, Ciencias de la Alimentación y Gastronomía, Facultad de Farmacia y Ciencias de la Alimentación, Instituto de Biomedicina (IBUB) e Instituto de Química Teórica y Computacional (ICTQBUB), Universidad de Barcelona, Campus Torribera, Santa Coloma de Gramenet, Spain; 4 Departamento de Ciencias Farmacéuticas y de la Salud, Facultad de Farmacia, Universidad San Pablo-CEU, Madrid, Spain

**Keywords:** Chemical biology, Drug discovery

## Abstract

Endothelial adenosine monophosphate-activated protein kinase (AMPK) plays a critical role in the regulation of vascular tone through stimulating nitric oxide (NO) release in endothelial cells. Since obesity leads to endothelial dysfunction and AMPK dysregulation, AMPK activation might be an important strategy to restore vascular function in cardiometabolic alterations. Here, we report the identification of a novel AMPK modulator, the indolic derivative IND6, which shows affinity for AMPKα1β1γ1, the primary AMPK isoform in human EA.Hy926 endothelial cells. IND6 shows inhibitory action of the enzymatic activity in vitro, but increases the levels of p-Thr^174^AMPK, p-Ser^1177^eNOS and p-Ser^79^ACC in EA.Hy926. This paradoxical finding might be explained by the ability of IND6 to act as a mixed-type inhibitor, but also to promote the enzyme activation by adopting two distinct binding modes at the ADaM site. Moreover, functional assays reveal that IND6 increased the eNOS-dependent production of NO and elicited a concentration-dependent vasodilation of endothelium-intact rat aorta due to AMPK and eNOS activation, demonstrating a functional activation of the AMPK–eNOS–NO endothelial pathway. This kinase inhibition profile, combined with the paradoxical AMPK activation in cells and arteries, suggests that these new chemical entities may constitute a valuable starting point for the development of new AMPK modulators with therapeutic potential for the treatment of vascular complications associated with obesity.

## Introduction

Obesity prevalence has increased over the past decades and is now a major public health problem worldwide. It is associated with an enhanced risk of developing cardiometabolic diseases such as hypertension, insulin resistance, type 2 diabetes mellitus, coronary artery disease, myocardial infarction, heart failure, and stroke^1^. Obesity involves changes in body composition as a consequence of an energetic imbalance in which caloric intake is higher than energy expenditure^1^. The AMP-activated protein kinase (AMPK) is a sensor of cellular energy status that is responsible for maintaining the energy balance after depletion of energy stores, switching off ATP-consuming anabolic pathways^2,3^.

Endothelial AMPK plays a key role in the regulation of vascular function through the activation of the PI3K-Akt-endothelial nitric oxide synthase (eNOS) pathway and stimulation of nitric oxide (NO) release in endothelial cells^4–6^. Obesity leads to AMPK dysregulation and endothelial dysfunction, which is the first step in the progression of cardiovascular disease^7^. We have shown that caloric restriction in young Zucker *fa/fa* rats has cardiovascular benefits by reducing endothelial dysfunction through AMPK–PI3K–Akt–eNOS activation associated to a reduction in blood pressure, plasma triglyceride levels, and cardiac hypertrophy^5^. AMPK activation might be thus an important strategy to restore vascular function in cardiometabolic alterations.

AMPK is a heterotrimeric Ser/Thr kinase of 1188 amino acids (~ 132 kDa), which is ubiquitously distributed. It is formed by three subunits: α (α1 and α2), β (β1 and β2) and γ (γ1, γ2 and γ3), which combine to give 12 different isoforms^8^. AMPKα is the catalytic subunit and contains a conventional kinase domain (αKD) located at the N-terminus of the protein, and a C-terminal domain required for interaction with the AMPKβ subunit. The C-terminal region of AMPKα subunit forms a globular domain around which the C-terminal region of the AMPKβ subunit is wrapped. The extreme terminus of the AMPKβ subunit then forms an interaction with the AMPKγ subunit, so that the AMPKβ subunit acts as the scaffold that bridges α and γ subunits. A carbohydrate-binding module (CBM), located within the central region of the AMPKβ subunit, forms a binding site for allosteric activators, termed the allosteric drug and metabolite (ADaM) binding pocket. The AMPKγ subunit contains four repeats in tandem of a structural module called cystathionine β-synthase (CBS) motif. Every pair of CBS repeats provides binding sites for the regulatory adenine nucleotides AXP (X = M, D, T)^9^.

The range of AMPK modulators has been gradually expanded over the last years^10,11^, covering from AMP mimetics, such as AICAR and C2, to ADP mimetics, such as O304, which is able to protect against pThr172 or pThr174 dephosphorylation in AMPKα2 and AMPKα1 isoforms, respectively, without allosteric activation of AMPK^12^. On top, AMPK activator drugs such as A769661, 991, PF-739, etc., directly activate AMPK targeting the ADaM site^13^. Strikingly, other AMPK modulators with unexpected mechanisms of action have been recently described. MT47-100^14^ is an allosteric AMPKβ2 inhibitor that simultaneously activates AMPKβ1, whereas SU6656^15^ paradoxically activates AMPK signaling by directly binding at the catalytic site. All these results reveal the complex regulation of this kinase, but at the same time offer the opportunity to be exploited in the search for drugs with novel mechanisms of action.

In this work, we describe novel indolic compounds as modulators of endothelial AMPK. For this purpose, the binding mode of these compounds has been assessed by combining molecular dynamics (MD) simulations, enzymatic and Surface Plasmon Resonance (SPR) assays, together with functional activation studies targeting AMPK, eNOS and Acetyl CoA Carboxylase (ACC) phosphorylation, as well as assessing NO production in human endothelial cells (EA. Hy926) and through vascular function of rat thoracic aorta. Our findings offer new possibilities for regulating endothelial AMPK, as well as exploring the therapeutic implications of this novel mechanism of action.

## Results

### Identification of IND6 as AMPKα1β1γ1 modulator

Within our drug discovery program focused on the search for novel AMPK modulators^16^, we combined surface plasmon resonance (SPR) assays with enzymatic activity studies with the aim to evaluate the ability of the indole derivatives (**IND6**, **IND7**, **IND8**, **IND11**) to bind to AMPKα1β1γ1 (Table 1).

**Table 1:** AMPK α1:β1:γ1 inhibitory activity and binding results measured by SPR (RU) of indole derivatives.

Compound	% Inhibition ± SD at 30 µM^a^	Binding 100 µM (RU)^b^
	AMPK α1:β1:γ1	
**IND6**	62.00 ± 2.77	13.3
**IND7**	39.42 ± 3.03	13.6
**IND8**	52.38 ± 2.51	14.6
**IND11**	23.46 ± 2.48	0.9

^a^Data is the mean ± standard deviation (SD) of two independent experiments. Positive control: A-769662: % activation ± SD at 30 µM: 394 ± 76.

^b^Binding (100 µM): Positive binding control: A-769662 (RU 31.7); Negative binding control: β-cyclodextrin (RU 1.6). Data is the mean ± SD of 1–6 independent experiments.

**Scheme 1:**
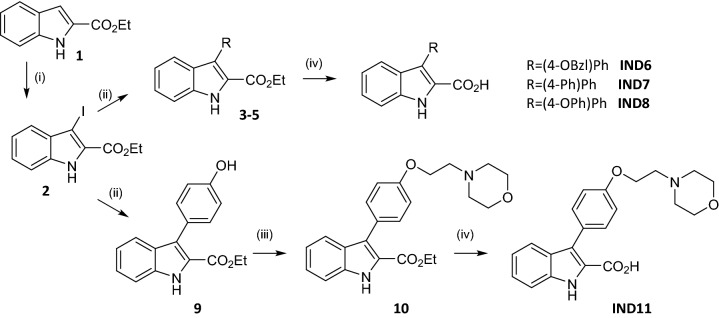
(i) iodosuccinimide, DMF, 0 °C, 1 h; (ii) toluene:ethanol:water:1,4-dioxane (1:3:6:10), R-B(OH)2, Pd (PPh_3_)_4_, K_2_CO_3_, 85 °C, 18 h; (iii) K_2_CO_3_, EtOH, 85 °C, 18 h (iv) KOH, EtOH, 100 °C, 18 h.

The synthetic route employed to prepare **IND6**, **IND7**, **IND8**, and **IND11** is depicted in Scheme 1. The iodation of intermediate **2** was obtained by reaction of 1H-indole 2-ethyl carboxylate (**1**) with N-iodosuccinimide in dry DMF. From the key intermediate **2**, aryl substituted indole derivatives **3–5** and **9** were prepared by reaction with the corresponding boronic acid derivative, Pd(dppf)Cl_2_ and K_2_CO_3_. Compound **10** was prepared by the reaction of ethyl 3-(4-hydroxyphenyl-1H-indole-2 carboxylate **9** with 4-(3-chloropropyl)morpholine hydrochloride. Further, KOH was employed to obtain carboxylic acid derivatives **IND6**, **IND7**, **IND8**, and **IND11**.

Compounds **IND6**, **IND7**, **IND8**, and **IND11** were screened for binding to the AMPKα1β1γ1 at a single concentration (100 µM) using the SPR technique. Abbott's product A-769662 was used as a positive binding control, and β-cyclodextrin, which binds preferentially to the AMPKβ2 isoform, as a negative binding control. Binding of **IND6**, **IND7**, or **IND8** to AMPKα1β1γ1 is slightly weaker than A-769662. **IND11** showed no response (0.9 RUs), suggesting the importance of the terminal aromatic ring present in the substituent at position 3 for AMPK binding, in contrast with the more polar character of the morpholine unit in **IND11**, which must be protonated at physiological pH (pK_a_ ~ 8.4).

In parallel, the effect of compounds **IND6**, **IND7**, **IND8**, and **IND11** on the enzymatic activity of AMPK was examined through a luminescent assay with the recombinant AMPK isoform α1β1γ1. This assay evaluates the enzymatic activity of AMPK to phosphorylate the SAMS peptide substrate, using A-769662 as positive control. All compounds in the series were found to reduce the activity of AMPK α1β1γ1 at 30 µM and therefore may be initially considered inhibitors (Table 1). At this single concentration the percentage of inhibition ranges from 39 to 62% for **IND6**, **IND7** and **IND8**, and it is less pronounced for **IND11** (23%). Based on these results, **IND6** was selected as representative compound to carry out a detailed evaluation of the biological effect on the enzyme activity.

### Competition assays of the indolic compound IND6

**Figure 1:**
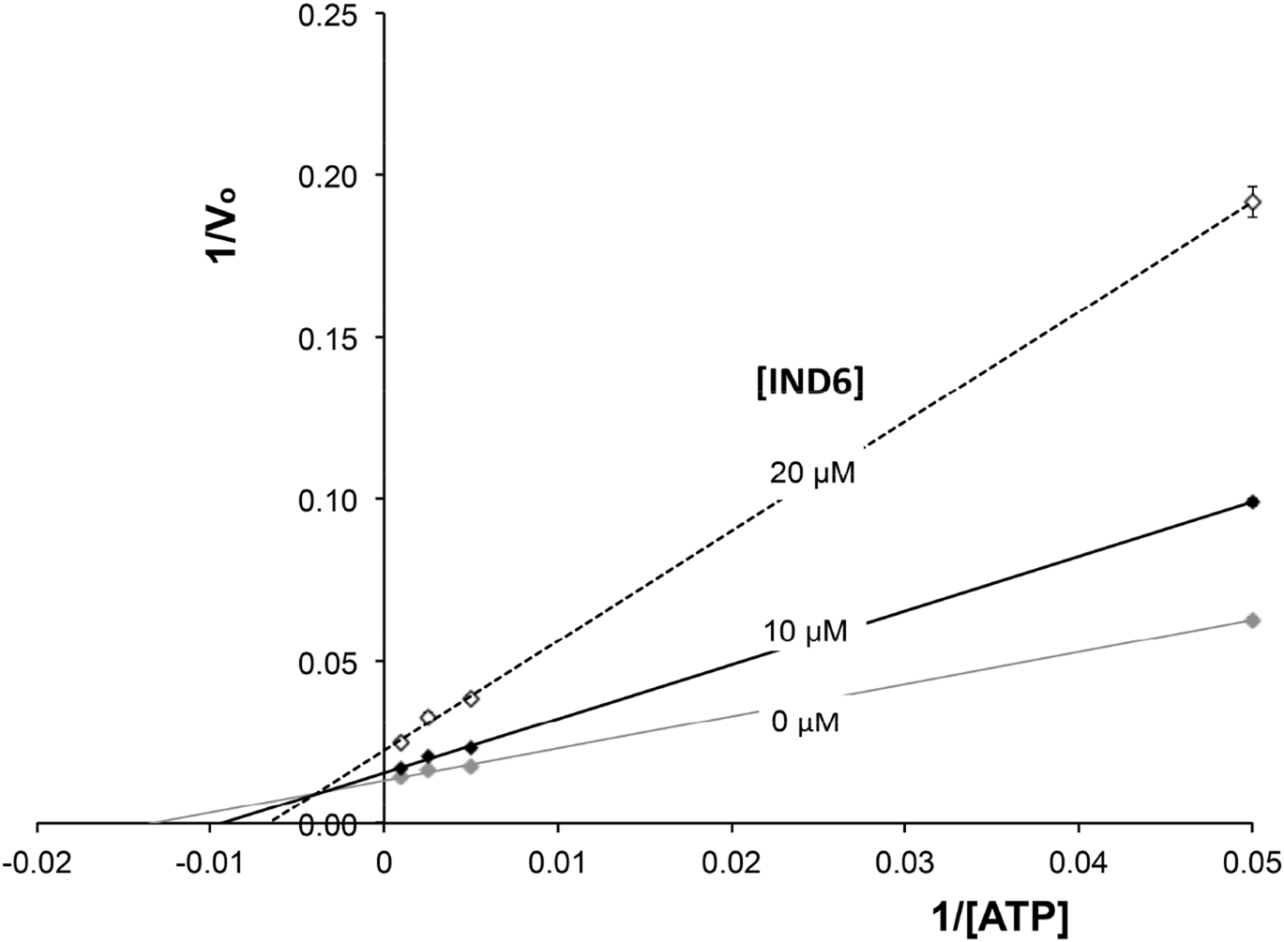
Lineweaver–Burk (double reciprocal) plot showing inhibition of AMPK (α1β1γ1) phosphorylation of SAMS peptide at two fixed IND6 concentrations (10 and 20 µM). Assays were performed in the presence of varying ATP concentrations (20–1000 µM) and the substrate concentration was kept constant at 0.2 µg/µL. Each point is the mean of two different experiments, each one analyzed in triplicate.

To investigate the inhibitory mechanism of **IND6**, dose–response assays were performed using different ATP concentrations (from 20 to 1000 µM) and two inhibitor concentrations (10 and 20 µM). The Lineweaver–Burk plot of enzyme kinetics is depicted in Fig. 1. The results suggest that **IND6** acts as a mixed-type inhibitor, as noted by the increase in the *K*_*s*_ for ATP and the decrease in *V*_*max*_ with increasing concentration of **IND6**. Let us note that this modality of enzyme inhibition was also reported previously for SBI-0206965^17^. Representation of the relationship between *Km/Vm* and the concentration of **IND6** led to inhibition constants *K*_*i*_ and $K^{\prime}_{i}$ of 6.9 and 27.1 µM, respectively. These values are 27- and 30-fold higher than the *K*_*i*_ and $K^{\prime}_{i}$ values determined for SBI-0206965, respectively (*K*_*i*_ = 0.26 µM; $K^{\prime}_{i}$ = 0.89 µM^17^). Overall, although both **IND6** and SBI-0206965 exhibit a mixed-type inhibition, which conceptually combines both competitive and uncompetitive inhibition, these results suggest that the inhibitory activity may reflect different mechanisms of action.

**Figure 2:**
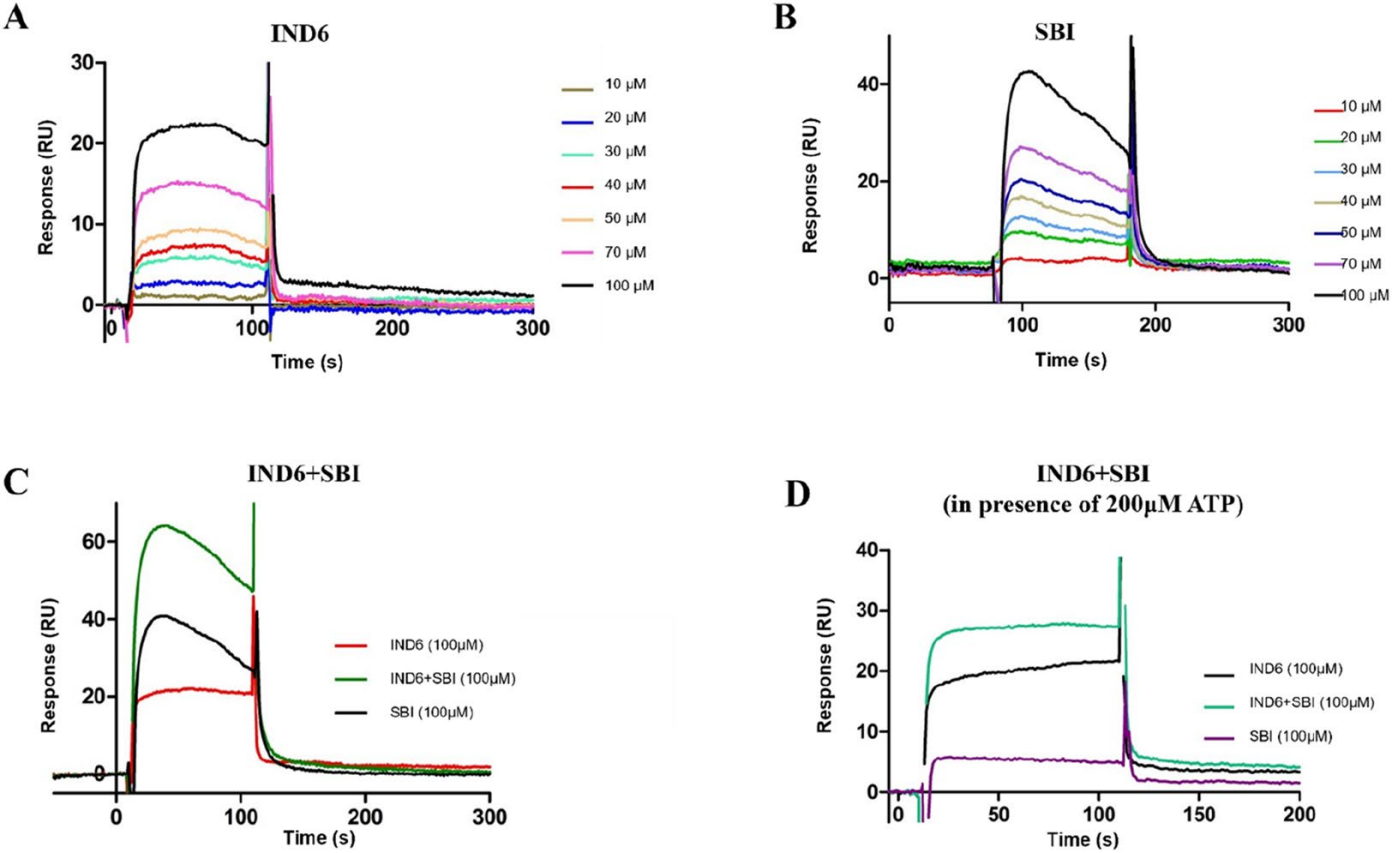
Typical sets of SPR binding curves for the interaction of AMPKα1β1γ1 with (**A**) IND6, (**B**) SBI-0206965, (**C**) SBI-0206965 plus IND6, and (**D**) SBI-0206965 plus IND6 in presence of ATP (200 µM). Experiments were performed increasing the ligand concentration from 10 to 100 μM (**A**,**B**) or at 100 μM for competitive experiments (**C**,**D**). The concentration series of ligand solutions were injected over the surfaces in duplicate or quadruplicate.

To further explore the **IND6** binding mode, SPR studies were performed at increasing concentrations (from 10 to 100 µM) of either SBI-0206965 or **IND6** (Fig. 2). The sensorgrams showed a progressive increase in the binding, which was much larger for SBI-0206965 in agreement with its stronger inhibitory potency (Fig. 2A,B). In a separate assay, **IND6** was injected in the presence of SBI-0206965, both at a 100 μM concentration. Under these conditions, the sensorgrams showed an additive effect between SBI-0206965 and **IND6** (Fig. 2C), suggesting that binding to AMPK might involve distinct binding sites. When the injection of SBI-0206965 and **IND6** was performed in the presence of a high ATP concentration (200 µM) (Fig. 2D), SBI-0206965 binding was notably reduced, whereas **IND6** binding was less sensible to the presence of ATP.

### IND6 promotes the phosphorylation and activation of AMPK and downstream targets in a concentration-independent manner

In order to examine the effect of IND6 in AMPK phosphorylation and some of its targets, such as eNOS and ACC, human endothelial cells of the EA Hy.926 line were treated with **IND6** at different concentrations (0.01, 0.1, 1, 10 and 100 μM). Untreated cells were used as control (CT). AICAR (at 5 mM), a known AMPK canonic activator, and 2-deoxyglucose (2-DG, at 1 mM), a caloric restriction mimetic, were used as positive controls. AICAR, once inside the cell, is phosphorylated by adenosine kinases and is converted to ZMP, an AMP mimetic, which binds the CBS sites in γ-AMPK^18^. On the other hand, 2-DG is a competitive inhibitor of glucose metabolism^14^ since it is phosphorylated by hexokinase to DG-PO_4_, which is trapped in the cell unable to undergo further metabolism^6,19^.

**Figure 3:**
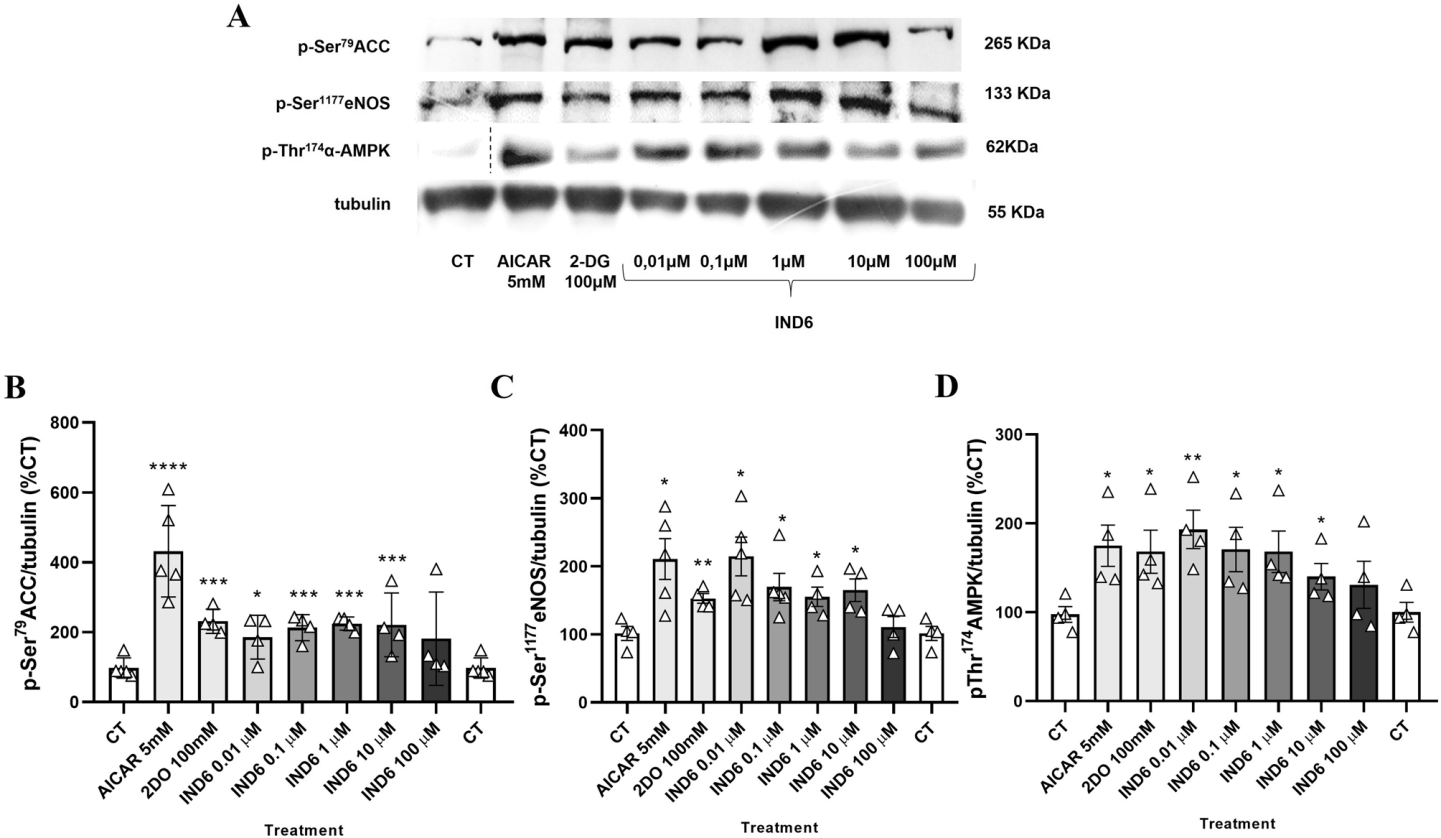
IND6 increases p-AMPK, p-ACC, and p-eNOS levels in a concentration-independent manner. (**A**) Representative immunoblots of p-Ser^79^ACC, p-Ser^1177^eNOS, p-Thr^174^AMPK and tubulin of EA Hy.926 human endothelial cells after the treatments (60 min for AICAR and IND6 and 15 min for 2-DG). (p-Thr^174^AMPK CT band was cropped from channel 9 of the gel to channel 1). (**B**) Bar chart representation of the densitometry of the immunosensing bands (WB) expressed as the percentage of p-Thr^174^AMPK/tubulin with respect to the control group. (**C**) Bar chart representation of the densitometry of the immunosensing bands (WB) expressed as the percentage of p-Ser^79^ACC/tubulin with respect to the control group. (**D**) Bar chart representation of the densitometry of the immunosensing bands (WB) expressed as the percentage of p-Ser^1177^eNOS/tubulin with respect to the control group. *p < 0.05; **p < 0.01. n = 4–5. Data are presented as means ± SEM.

**IND6** significantly increased the level of p-Thr^174^AMPK at concentrations of 0.01, 0.1, 1 and 10 µM in a concentration-independent manner (Fig. 3A,B). The effect was comparable to the enhancement elicited by AICAR at 5 mM and 2-DG at 1 mM. Since the increased phosphorylation of AMPK is in contrast with the mixed-inhibitor features observed in the enzymatic assays, we determined levels of p-Ser^79^ACC and p-Ser^1177^eNOS, which are well-known targets of AMPK in the endothelium^20,21^. **IND6** significantly increased the levels of both p-Ser^79^ACC/tubulin and p-Ser^1177^eNOS/tubulin at concentrations of 0.01, 0.1, 1 and 10 µM in a concentration-independent manner (Fig. 3A,C,D), suggesting that **IND6** promotes a functional activation of AMPK, this effect being nevertheless less apparent at the highest concentration of 100 µM.

### Binding mode of IND6 to AMPK

**Figure 4:**
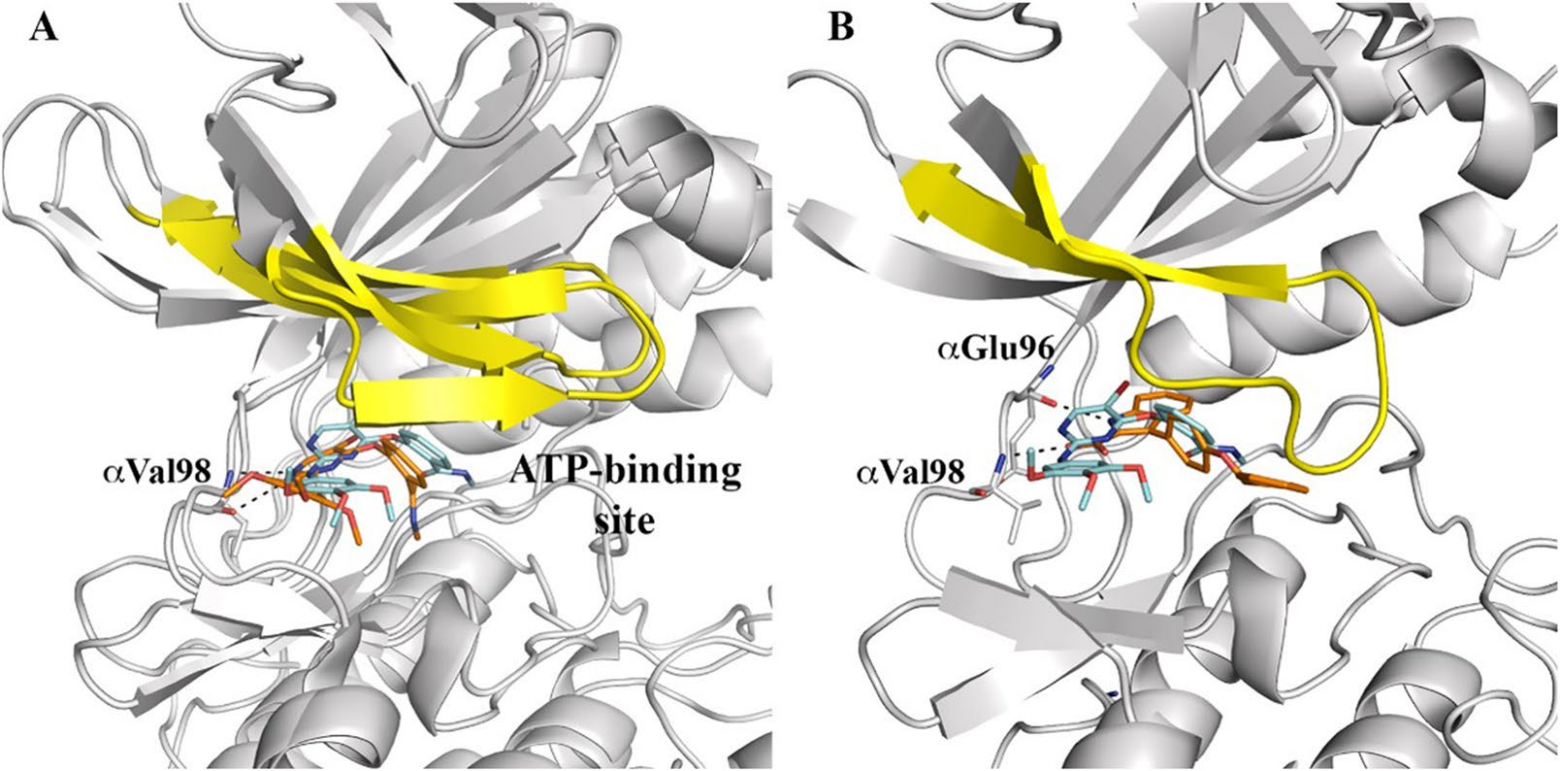
Representation of the binding mode of SBI-020695 and IND6 to the ATP-binding site of AMPK obtained from Molecular Dynamics simulations. (**A**) Superposition of the X-ray crystallographic structure of AMPK bound to SBI-020695 and a representative snapshot taken from the MD simulations. (**B**) Representative snapshot of the MD simulation run for the AMPK–IND6 complex. The P-loop is highlighted in yellow. SBI-020695 and IND6 are shown in sticks. C atoms colored in cyan and orange shown the X-ray and final position of the ligand in MD simulation, respectively. Hydrogen atoms have been removed for the sake of clarity.

Due to the mixed-type inhibition of **IND6**, we investigated the binding to the ATP-binding site using Molecular Dynamics (MD) simulations. Four independent simulations were run for the AMPKα1β1γ1 complexes with **IND6** and with SBI-020695, which was used as reference system. The X-ray structure of the AMPK–SBI-020695 complex (PDB entry 6BX6) revealed that SBI-0206965 occupies a pocket located between the N- and C-lobes and the hinge region of the enzyme, overlapping with the binding site of compound C, which is a competitive inhibitor of AMPK^22^. The results obtained from the different MD simulations showed a consistent picture, where SBI-0206965 remains stably bound in the ATP-binding pocket in all simulations (Fig. 4). In particular, binding is assisted by two hydrogen bonds between SBI-0206965 and the main chain of αVal98, with distances (averaged for the four MD simulations) of 3.2 ± 0.2 Å and 2.9 ± 0.1 Å between the pyrimidine nitrogen and exocyclic nitrogen of the inhibitor and the amide NH and carbonyl oxygen of αVal98, respectively. Furthermore, the ligand is enclosed in the hydrophobic pocket shaped by residues αLeu 24, αVal32, αIle79, αMet95, αLeu148 and αAla158.

The competitive binding mode of **IND6** to the ATP-binding pocket was guided by the superposition with both staurosporine, SBI-020695 and compound C, taking advantage of their X-ray structures (PDB entries 4ZHX, 4CFE, 4CFF, 6BX6 and 3AQV; see Supplementary Material), which revealed the formation of hydrogen bonds between these compounds with the hinge region of the kinase, particularly involving residues αVal96 and αGlu94 (αVal98 and αGlu96 in AMPKα1).

The MD simulations performed for the AMPK–**IND6** complex revealed larger fluctuations of the ligand in the binding pocket compared to SBI-020695 (Fig. 4A). This trait can be attributed to the flexibility of the benzyloxy moiety as well as to the non-planarity of the central benzene ring relative to the indole ring, enhancing also the fluctuations of the P loop (Fig. 4B). This is also reflected in the hydrogen bond distances formed between the indole NH group of **IND6** with the carbonyl oxygen of αVal98 (average distance of 3.4 ± 1.1 Å), and the carboxylate oxygen of **IND6** with the NH group of αGlu96 (average distance of 3.7 ± 0.9 Å), which are larger than those formed by SBI-020695. Overall, these traits agree with the 27-fold lower potency of **IND6** relative to SBI-020695 (see above).

**Figure 5:**
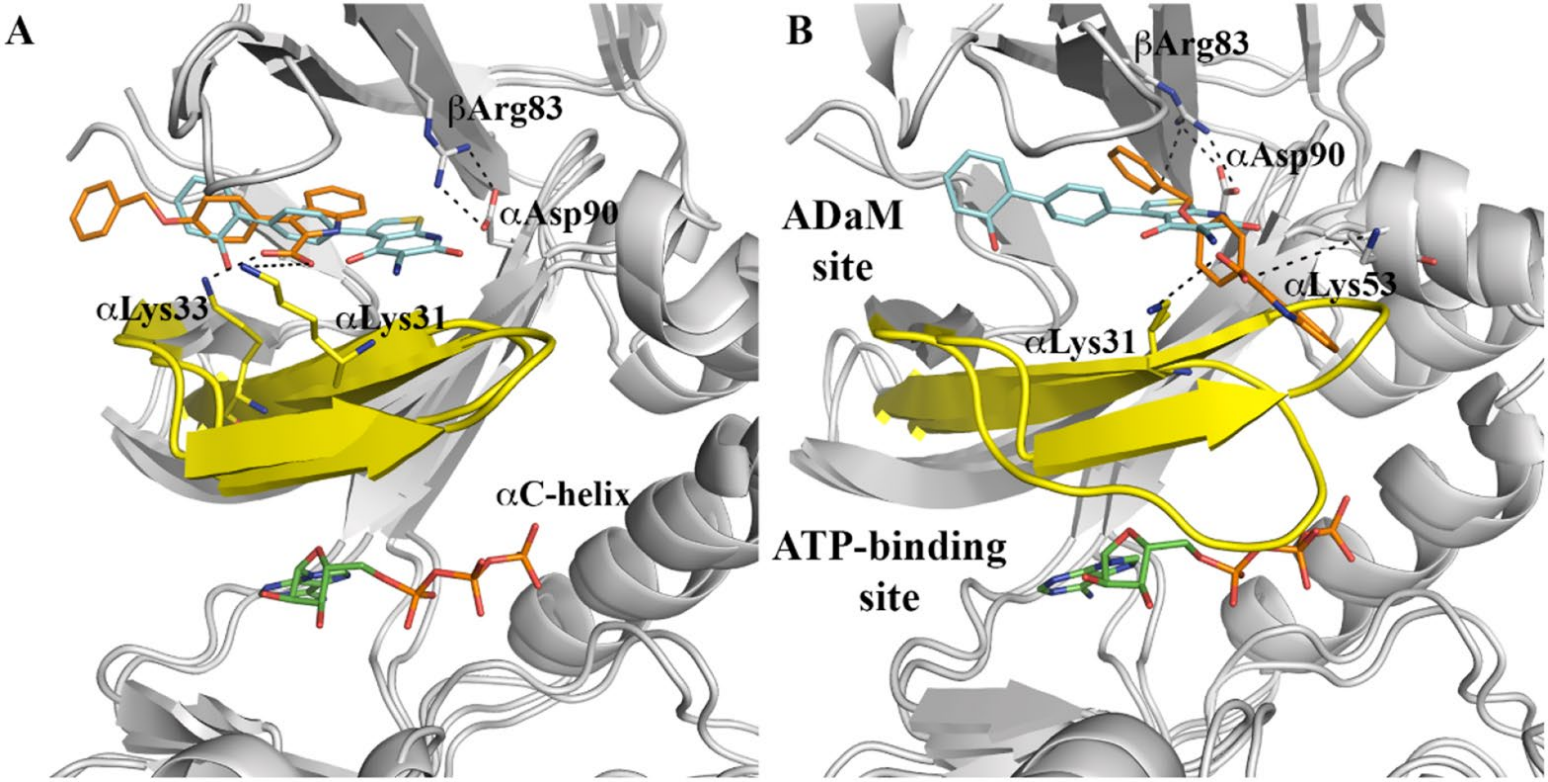
Superposition of the X-ray structure of the ternary complex formed by AMPK bound to A-769662 and ATP (C atoms in cyan and green, respectively; taken from^24^), and a representative snapshot of IND6 (C atoms in orange) bound to the ADaM site taken from the Molecular Dynamics simulation. (**A**) Activating binding mode. (**B**) Non-competitive binding mode. The P-loop is shown in yellow. Selected interactions with protein residues are indicated as dashed lines. Hydrogen atoms have been removed for the sake of clarity.

**Figure 6:**
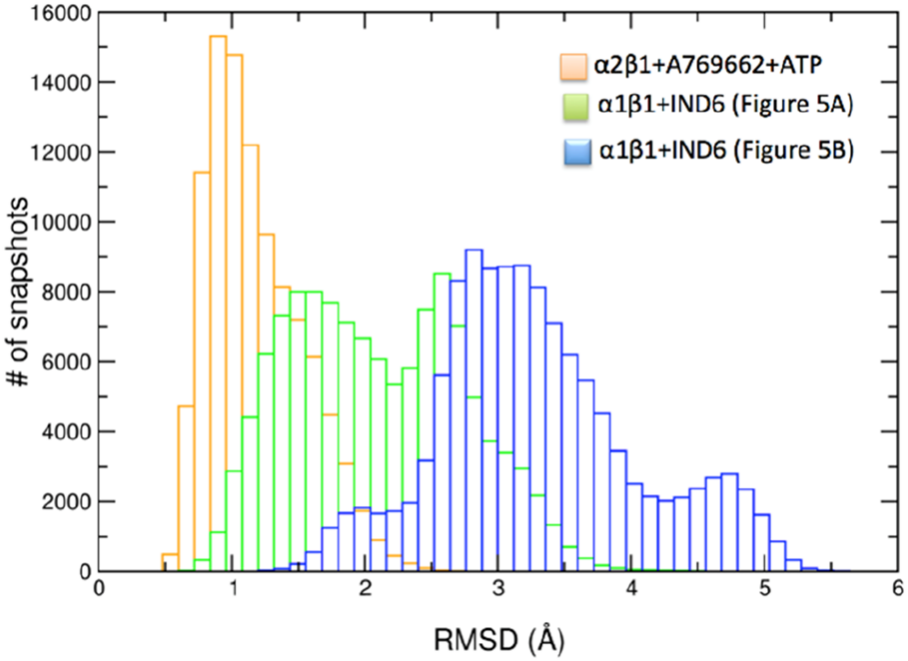
Distribution of the positional deviation (RMSD; Å) of the backbone atoms that shape the ATP-binding along the trajectories run for the two binding modes of IND6 bound to AMPKα1β1 (shown in Fig. 5A,B), and for the ternary complex between A-769662, ATP and AMPKα2β1 (data taken from^24^).

Additional MD simulations were also performed to examine the binding of **IND6** to the ADaM site, which mediates the activation effect played by several small molecules, such as A-769662^23^. The ligand was oriented taking advantage of the close alignment exhibited by activators such as A-769662, 991 and SC4 (see Supplementary Material). These studies showed that **IND6** may adopt two distinct binding modes (Fig. 5). In one case, **IND6** is deeply bound into the hydrophobic cavity of the ADaM site, and the carboxylate group forms salt bridge interactions with the protonated amino groups of αLys31 and αLys33 (average distances of 3.0 ± 0.4 and 3.5 ± 0.9 Å). It is worth noting that the top of the P-loop points to the N-terminus of the αC-helix, leaving the ATP-binding site accessible for the binding of ATP. Indeed, a significant fraction of the conformations sampled by the P-loop superpose well with the conformations adopted in the ternary complex formed by AMPK bound to A-769662 and ATP^24^ (Fig. 6). This suggests that **IND6** might mimic the role of A-769662 in this binding mode.

In the other binding mode, **IND6** protrudes from the ADaM site toward the αC-helix, sitting on the top of the P-loop (Fig. 5B). This binding mode is assisted by electrostatic interactions between the carboxylate group and the protonated residues αLys31 and αLys53 (average distances of 4.9 ± 0.9 and 5.3 ± 1.1 Å), and a cation-π interaction between βArg83 and the benzyloxy ring of **IND6** (average distance of 4.0 ± 0.5 Å). Remarkably, this binding mode imposes a structural distortion of the P-loop, which occludes the ATP-binding site (Figs. 5B and 6), making it unable to accommodate ATP. Therefore, this binding mode might explain the non-competitive mechanism of the mixed-type inhibition.

### IND6 promotes eNOS-dependent NO production in human endothelial cells with a major potency than AICAR does

**Figure 7:**
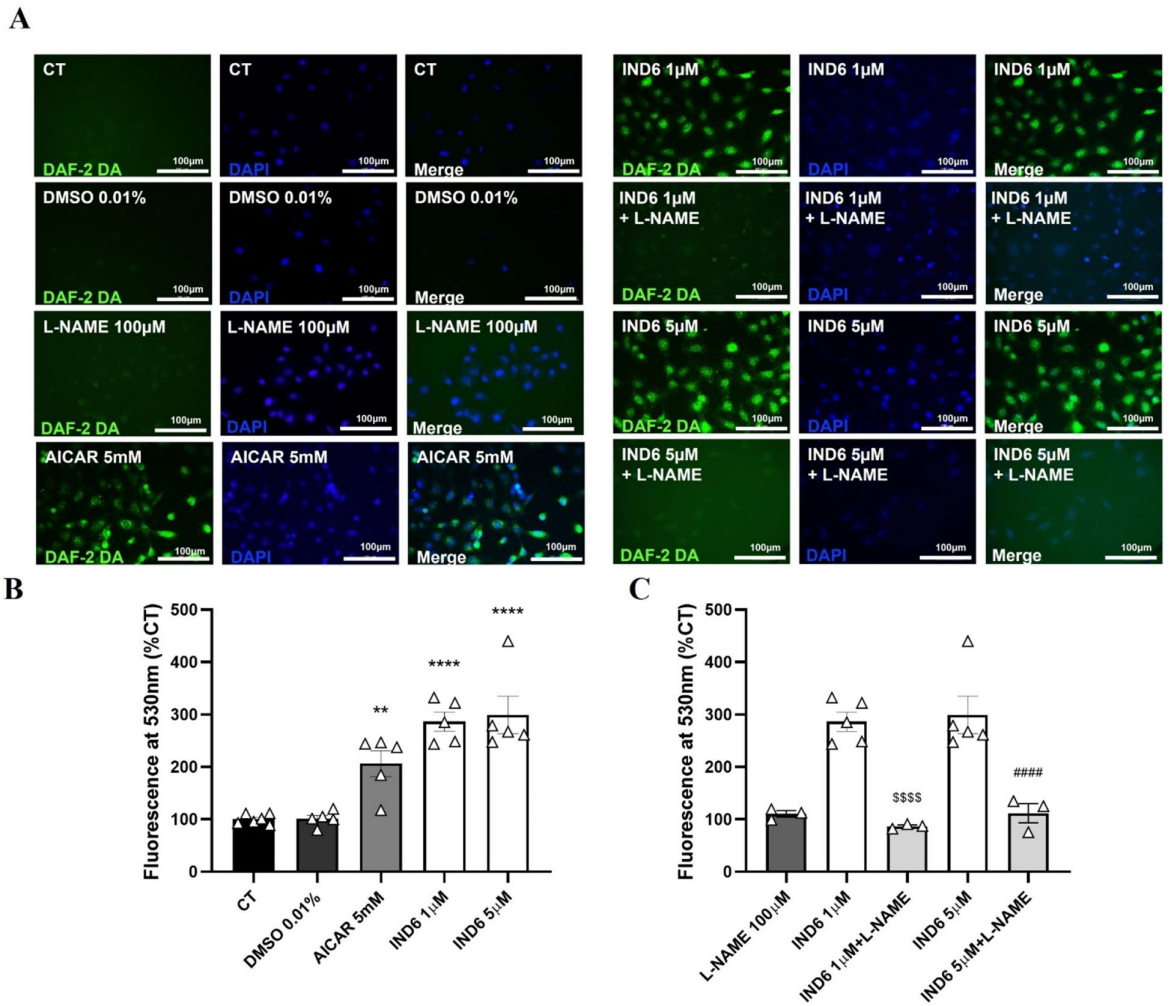
IND6 promotes an increase in DAF-2T fluorescence in human endothelial cells. (**A**) Representative fluorescence microscopy images of NO fluorescent indicator (DAF-2D incubation, first column left and right, green) and DAPI (second column left and right, blue) in cultured EA.Hy926 endothelial cells in basal conditions (CT) and after 60 min treatment with DMSO 0.01%, eNOS inhibitor L-NAME (100 μM), AMPK activator AICAR (5 mM), IND6 (1 and 5 μM) and IND6 (1 and 5 μM) + L-NAME (100 Μm). (n = 3–5). (**B**) Bar chart of the quantification of the intensity of green fluorescence (530 nm.) by densitometry represented as the percentage with respect to the control cells. **p < 0.01 vs. CT and ****p < 0.0001 vs. CT; n = 3–5. (**C**) Bar chart of the quantification of the intensity of green fluorescence (530 nm) by densitometry represented as the percentage with respect to the control cells. ^$$$$^p < 0.0001 vs. IND6 1 μM and ^####^p < 0.0001 vs. IND6 5 μM; n = 3–5. The data are presented as means ± SEM.

To determine whether the increase in p-Thr^174^AMPK and p-Ser^1177^eNOS elicited by **IND6** translates to^5,21^ an increment of NO production, NO levels were determined in human endothelial cells EA Hy.926 in presence of 1 and 5 µM **IND6**. Untreated cells were used as a control (CT). Cells treated with DMSO 0.01% [maximum concentration of solvent for **IND6** (5 µM)] were used to exclude cytotoxic effects. AICAR (at 5 mM) was used as a positive activation control (see “IND6 promotes the phosphorylation and activation of AMPK and downstream targets in a concentration-independent manner”). AICAR induces an increase in fluorescence of DAF-2T, which indirectly represents the release of NO. **IND6** (at both 1 and 5 µM) induces a similar increase in DAF-2T fluorescence as AICAR, but at 5000 × lower concentrations (Fig. 7A,B). To confirm that the increase in DAF-2T fluorescence is a real consequence of eNOS activation, pretreatments with the eNOS inhibitor L-NAME (100 µM), were performed in absence or in presence of **IND6** (1 and 5 µM). L-NAME abolishes **IND6**-induced increment of DAF-2T fluorescence indicating that NO increase is a consequence of eNOS activation (Fig. 7A,C).

### IND6 promotes a concentration-dependent vasodilation in rat aorta that is mediated by the AMPK–eNOS–NO pathway

**Figure 8:**
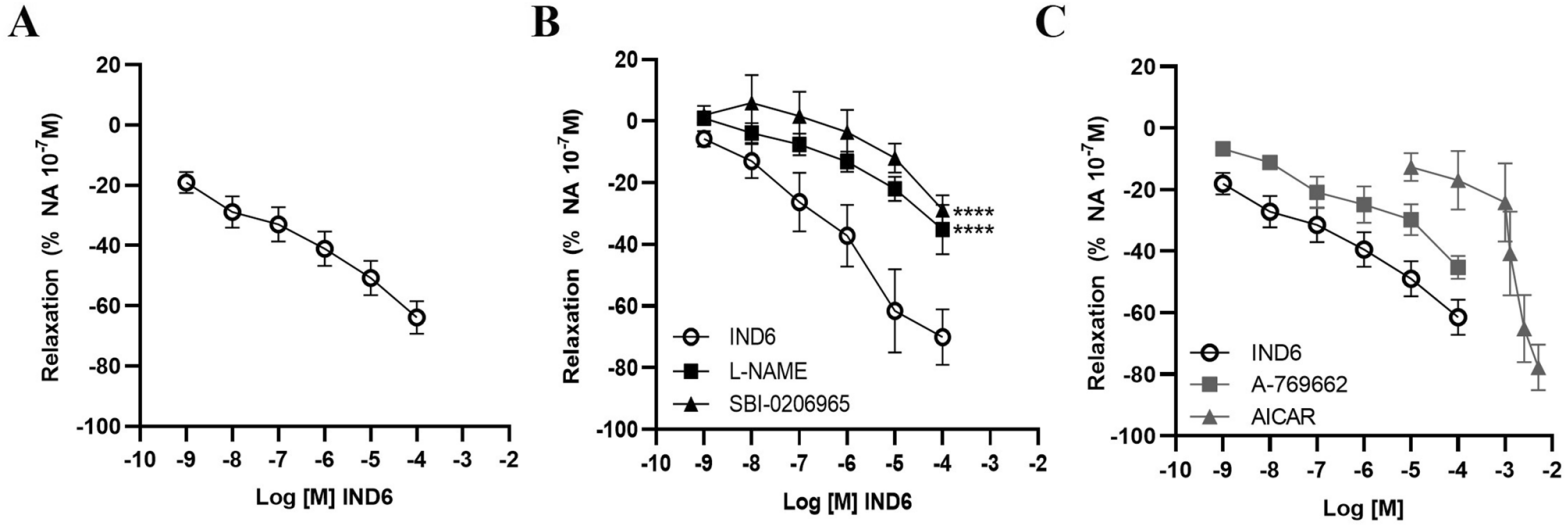
IND6 promotes an AMPK–eNOS-dependent vasodilation of rat thoracic aorta. Concentration–response curves in rat aortic rings with intact endothelium precontracted with noradrenalin 10^−7^ M in response to: (**A**) IND6 10^−9^–10^−4^ mol/L; (**B**) IND6 10^−9^–10^−4^ mol/L in presence of the eNOS inhibitor L-NAME (10^−4^ M) or in presence of the AMPK inhibitor SBI-0206965 (10^−4^ M): (**C**) AICAR 10^–5^–8 × 10^–3^ mol/L, A-769662 and IND6 10^−9^–10^−4^ mol/L. The data are represented as means ± SEM; n = 6 rats.

To confirm the activation of the AMPK–eNOS–NO pathway by **IND6**, vascular function was assessed in endothelium-intact thoracic aorta of Wistar rats. A vasodilatory response to **IND6** was observed in a concentration-dependent manner (10^−9^–10^−4^ M) in artery segments precontracted with a single dose of NA 10^−7^ M (Fig. 8A). Relaxation elicited by **IND6** was significantly reduced in artery segments preincubated with SBI-0206965 (10^−4^ M) or L-NAME (10^−4^ M) (Fig. 8B), which are inhibitors of AMPK and eNOS, respectively. Furthermore, concentration–response curves were also performed with A-769662 (10^–9^–10^−4^ M) or AICAR (10^–5^–8 × 10^−3^ M) to compare their effect with the one promoted by **IND6** (Fig. 8C). Since the three of them elicited a concentration-dependent vasodilation, their pharmacological efficacy (E_max_) and potency (EC_50_) were calculated. Although the three compounds had a similar E_max_, **IND6** resulted as potent as A-769662 but significantly more potent than AICAR (Table 2).

**Table 2:** Efficacy (E_max_) and potency (EC_50_) of **IND6**, A-769662 and AICAR as vasodilators in rat aorta measured with organ bath vascular function. ***p-value < 0.001 vs. AICAR; n = 6 rats.

Compound	E_max_	EC_50_
IND6	− 63.9 ± 11.5% NA 10^−7^ M	2.2 × 10^–6^ M***
A-769662	− 45.3 ± 2.6% NA 10^−7^ M	4.6 × 10^–5^ M
AICAR	− 77.8 ± 9.1% NA 10^−7^ M	1.4 × 10^–3^ M

## Discussion

The results presented in this study point out that the novel indolic derivatives appear to act as paradoxical activators of the endothelial AMPK α1β1γ1, although they exhibit a mixed-type inhibition in the enzymatic assays.

Dysregulation in the AMPK signaling pathway in over-nutrition and obesity contributes to the development of metabolic disorders and endothelial dysfunction^1^, which is considered the first step in the progression of cardiovascular disease^2^. Reduced endothelial AMPK phosphorylation leads to down-regulation of the PI3K-Akt-eNOS pathway together with low rates of NO synthesis^5,7^. Contrarily, activation of endothelial AMPK restores impaired endothelial function and normalizes systolic blood pressure through the stimulation of the PI3K-Akt-eNOS pathway^5,25^. In this context, identification of new chemical entities that can activate endothelial AMPK could be of significant interest for the treatment of obesity-related disorders. Starting with the use of SPR techniques, we selected **IND6**, which exhibits one of the best affinity values against recombinant AMPKα1β1γ1 (RU 13.2, 100 µM). In parallel, the AMPK α1β1γ1 enzymatic activity was assessed by means of a luminescent assay, which revealed inhibitory activity values in the micromolar range against all tested compounds (Table 1). Hence, **IND6** was subjected to enzymatic kinetic analysis to examine its competition with ATP. We varied both ATP and **IND6** concentrations with a constant concentration of the peptide substrate used in the enzymatic reaction. The double reciprocal plot of data (Fig. 1) indicated that **IND6** behaves as a mixed-type AMPK inhibitor. A similar inhibition mode has been recently reported for SBI-0206965, which was described as type IIb AMPK inhibitor^17^. Furthermore, SPR sensorgrams showed that **IND6** and SBI-0206965 presented an additive effect on the binding to AMPK, suggesting that they may bind at different binding sites. Moreover, when we performed the same experiments in the presence of a high ATP concentration (200 µM), there was a significant reduction in the ability of SBI-020696 to bind to AMPK, while the affinity of **IND6** for AMPK remained at large extent unchanged. All these results suggest that **IND6** and SBI-020696 may bind the ATP-binding site, thus leading to competitive inhibition of the enzyme, but also suggest that **IND6** may regulate the AMPK activity through binding to an additional pocket.

The biological effect of **IND6** may be explained from the distinct binding modes observed for **IND6** in the ADaM site, and the drastic influence exerted on the structural conformation of the P-loop. Thus, the similar arrangements observed for the P-loop when **IND6** is deeply inserted into the ADaM site and in the X-ray structure of the AMPK bound to A-769662 suggest that **IND6** may mimic the activating role attributed to this latter compound. Nevertheless, the structural distortion of the P-loop caused by the alternative binding mode, where **IND6** protrudes from the ADaM site, might explain the non-competitive component of the mixed-type inhibition, in conjunction with the direct competition exerted by **IND6** upon binding to the ATP-binding site. At this point, let us remark that the adoption of two partially overlapping binding modes at the ADaM site may be facilitated by the lack of direct interactions between **IND6** and α-Asp90, in contrast to A-769662, which was found to form a hydrogen bond interaction with this residue in our previous studies of the AMPKα2β1–A769662 complex^24^. In fact, previous experimental studies demonstrated that the interaction between βArg83, αAsp90 and A-769662 is crucial for the enzyme activation^10^.

Despite the mixed-type inhibition, cellular assays showed that **IND6** promotes AMPK phosphorylation (% p-AMPK/tubulin vs*.* CT) in the human endothelial cell line, EA Hy.926, which expresses the α1β1 isoform. This suggests a paradoxical activation of AMPK similar to the one described for the indolic compound SU6656, which seems to promote AMPK’s LKB1 dependent phosphorylation^15,26^. In any case, the activation of AMPK by IND6 is functional since it translates to ACC, an ubiquitous AMPK target^20^, as well as to eNOS and NO release. The inhibition elicited by SBI-0206965 on the concentration-dependent relaxation induced by **IND6** in arterial rings confirms specific activation of vascular AMPK, whereas its inhibition by L-NAME confirms activation of endothelial eNOS. This is in accordance with the AMPK-dependent eNOS activation described in endothelial cells^5,21^, as well as in arteries associated to a reduction in blood pressure, plasma triglyceride levels, and cardiac hypertrophy^5^. Activation of eNOS by AMPK is a well-described pathway, although an attenuated NO production in response to AMPK activation has also been reported^27^.

The efficacy of **IND6** to promote AMPK stimulation is comparable to AICAR (5 mM), 2-DG (1 mM) or A-769662 (0.1 mM). However, the potency of IND6 is in the micromolar range, one and three orders of magnitude higher than A-769662 and AICAR, respectively, as clearly observed in the relaxation curves. To note, the apparent discrepancy between the concentrations of **IND6** tested in the in vitro assays with recombinant proteins (SPR or inhibition studies) and the active concentrations observed in cell culture, which are much lower than in the former case. This could be explained by the synergic effect of the intracellular machinery, which leads to signal amplification^28^.

In summary, **IND6** binding profile provides a basis to rationalize the activating behavior of **IND6** in EA Hy.926 cells and arteries by increasing AMPK activity in a functional manner, as demonstrated by the increment in both ACC and eNOS phosphorylation, two well-known targets of p-Thr172/174AMPK^20,21^. Moreover, this study shows that **IND6** increases NO levels in both endothelial cells and arteries, demonstrating again a functional activation of AMPK. Our findings provide evidence that **IND6** holds potential as treatment of vascular complications associated with obesity, where intracellular ATP levels are high due to the energy surplus and AMPK activity is reduced^5^. AMPK activation by **IND6** might be thus an important strategy to restore vascular function in cardiometabolic alterations.

## Methods

### Chemistry

All reagents were of commercial quality. Solvents were dried and purified by standard methods. Analytical TLC was performed on aluminum sheets coated with a 0.2 mm layer of silica gel 60 F254. Silica gel 60 (230–400 mesh) was used for flash chromatography. Analytical HPLC–MS was performed on Waters equipment coupled to a single quadrupole ESI–MS (Waters Micromass ZQ 2000) using a reverse-phase SunFire C18 4.6 × 50 mm column (3.5 μm) at a flow rate of 1 mL/min and by using a diode array UV detector. Mixtures of CH_3_CN and H_2_O were used as mobile phase (gradient of 15–95% of acetonitrile in water, as indicated in each case). HRMS (EI+) was carried out on Agilent 6520 Accurate-Mass Q-TOF LC/MS equipment. NMR spectra were recorded on a Bruker-AVANCE 300, a Varian-INOVA 400 and VARIAN SYSTEM-500 spectrometer. Melting points were determined on a Mettler MP70 apparatus and are uncorrected.

For experimental details, description of all synthetic intermediates, and characterization of final compounds see the Supplementary Material.

Synthesis of IND6: 3-(4-(Benzyloxy)phenyl)-1H-indole-2-carboxylic acid: To a solution of ethyl 3-(4-(benzyloxy)phenyl)-1H-indole-2-carboxylate (120 mg, 0.30 mmol) in EtOH (30 mL), KOH (30 mg, 1.10 mmol) dissolved in H2O (2 mL) was added. After stirring at 100 °C for 18 h, HCl 1 N (2 mL) was added. The resultant solid was filtrated and dried under reduced pressure to give 20 mg (18%) of IND6. HPLC (SunFire): tR = 9.51 min (gradient: 15–95% of acetonitrile in water). MS (ES, positive mode): 344 (M+H)+. Mp 194–196 °C. 1H NMR (300 MHz, DMSO-d_6_) δ 12.79 (s, 1H), 11.73 (s, 1H), 7.55–7.22 (m, 10H), 7.12–7.01 (m, 3H), 5.16 (s, 2H). 13C NMR (75 MHz, DMSO-d6) δ 163.2; 157.7; 137.6; 136.3; 131.9; 128.8; 128.2; 128.1; 127.4; 126.6; 125.0; 123.6; 122.1; 120.9; 120.5; 114.4; 112.9; 69.6. HRMS (EI+) m/z ([M]+) calcd for C_22_H_17_NO_3_ 343.12175; found 343.12179.

### Identification of the AMPK isoform expressed by human endothelial cells EAHy.926 by proteomics

In order to choose the AMPK isoform to carry out the kinase assay and affinity studies, we determined the isoform mainly expressed in EA Hy.926 cells. Cell lysates were analyzed in the proteomics unit of the UCM (CAI Técnicas Biológicas). For this purpose, protein precipitation and digestion with trypsin gel was carried out. Then, LC–MS/MS (Q-EXACTIVE) analysis of triptic peptides was performed (massive analysis: Shotgun and directed analysis: Parallel Reaction Monitoring, PRM), obtaining an m/z MS–MS spectrum that was used for the subsequent database search and identification based on the results. After comparing the information obtained with the data dumped in the databases, it was concluded that the most abundant isoform of AMPK in these cells is AMPKα1β1 with 99.99% accuracy. Although the identification of the subunit γ was not conclusive, we chose the recombinant isoform AMPKα1β1γ1 for the affinity and kinase assay studies.

### Binding studies by surface plasmon resonance (SPR)

SPR experiments were performed at 25 °C with a Biacore X-100 apparatus (Biacore, GE) in HBS-EP (10 mM Hepes, 150 mM NaCl, 3 mM EDTA), with 2% de DMSO, 0.05% Tween 20 and 200 µM ATP when was required, at 25 °C. The protein AMPk was inmobilized on a CM5 sensor chip (Biacore, GE) following standard amine coupling method^29^. The carboxymethyl dextran surface of the flow cell 2 was activated with a 7-min injection of a 1:1 ratio of 0.4 M EDC and 0.1 M NHS. The protein was coupled to the surface with a 300 s injection at several dilutions at 40 µg/mL in 10 mM sodium acetate, pH 5.0. The unreacted N-hydroxysuccinimide esters were quenched by a 7-min injection of 0.1 M ethanolamine-HCl (pH 8.0). The levels of immobilization were around 1000 RUs. Flow cell 1 treated as a flow cell 2 (amine coupling procedure) without protein was used as a reference. Prior to use 10 mM stock solutions of compounds were diluted several times until 100 µM final concentration in the running buffer. Typically, a series of different compounds was injected onto the sensor chip a flow rate of 30 µL/min for a period of 100 s followed by a dissociation period of 200 s. After the dissociation process an extrawash treatment was made over the flow cells with a 50% DMSO solution. No regeneration was needed. For competition measurements between AMPK and different compounds the concentrations used in the mixture were 100 µM for each one. Sensorgrams data were double-referenced and solvent corrected using the Biaevaluation X-100 software (Biacore, GE).

### Kinase assay

The AMPK (α1/β1/γ1) Kinase Enzyme System from Promega (Catalog number V1921) was used to screen AMPK inhibitors following the ADP-Glo™ Kinase Assay (Catalog number V9021). The assays were performed in 96-well plates (final volume 20 µL), the assay buffer contains 40 mM Tris, 7.5, 20 mM MgCl2, 0.1 mg/mL BSA and 50 μM DTT. 4 µL of inhibitor was added to each well (final concentration of DMSO did not exceed 1%), followed by 8 µL of enzyme (30 ng), after 5 min incubation at R/T, 8 µL of ATP (150 µM final concentration) and SAMStide (0.2 µg/µL) were added and incubate 60 min at room temperature, then ADP-Glo™ reagent (20 μL) was added allowing to incubate for 40 min at room temperature. Behind the incubation, the kinase detection agent (40 μL) was added and incubated for 30 min at room temperature. Finally, the luminescence was recorded using a FLUO star Optima (BMG Labtechnologies GmbH, Offenburg, Germany) multimode reader. The inhibition activities were calculated based on the maximum activity, measured in the absence of inhibitor.

For competition assays, the experiments were carried out at four different concentrations of ATP (20–1000 µM) in the absence or presence of the inhibitors, at two concentrations. The results were presented as double reciprocal Lineweaver–Burk plots (1/V vs 1/[ATP]).

### Molecular modelling simulations

Molecular Dynamics (MD) simulations were used to examine the binding mode of SBI-020695 and IND6 to AMPK, considering the binding to both the ATP-binding site and the ADaM site, which is implicated in the enzyme activation by small molecules, such as A-769662. The X-ray structure of AMPK in the PDB entry 6C9J^30^, which consists of chains α_1_, β_1_ and γ_1_, was utilized to build up the protein, and the position of SBI-020695 in the ATP-binding pocket was defined after superposition with the X-ray structure 6BX6^31^, which contains SBI-020695 bound to AMPK (isoform α2). To generate the IND6-bound complexes, SBI-020695 and A-769662 (PDB entry 4CFF) were replaced by IND6 in order to build up the simulated systems with IND6 in the ATP-binding and ADaM sites, respectively. Following our previous studies^23^, the γ-subunit was not considered in MD simulations because it does not participate in the inhibition process by SBI and IND6. Moreover, due to the lack of information of C-terminal tails of α and β subunits in the X-ray structure, these parts were not treated in our simulated systems. Finally, Thr174 was simulated in the phosphorylated form (pThr174).

Simulations were performed using the AMBER18 package^32^ and the Amber ff99SB-ildn force field^33^ for the protein, whereas the ligand (SBI, IND6) was parameterized using the GAFF force field in conjunction with restrained electrostatic potential-fitted (RESP) partial atomic charges derived from B3LYP/6-31G(d)^34^ calculations. The two simulated systems were immersed in an octahedral box of TIP3P water molecules^35^. The final systems contained around 370 residues, the ligand, around 26,000 water molecules, and one/two Na^+^ atom for the complexes with SBI/IND6, which were added to maintain the neutrality of the simulated systems.

Simulations were done in the NPT ensemble for equilibration and NVT for MD productions. The simulations for SBI and IND6 were performed for 4 independent replicas. The minimization of the two systems was performed refining the position of hydrogen atoms in the protein (2000 cycles of steepest descent algorithm followed by 8000 cycles of conjugate gradient), and subsequently of the whole system (4000 cycles for steepest descent and 1000 cycles of conjugate gradient). Then, the temperature of the system was gradually increased from 100 to 300 K in 5 steps (50 ps each) using the NVT ensemble, followed by an additional 5 ns step performed in the NPT ensemble to equilibrate the density of the system. In this process, restraints were imposed to avoid artefactual changes in the hydrogen bonds between the ligand with Val98, as well as between pThr174 and Arg140. Production MD simulations were run for 250 ns per replica, leading to a total simulation time of 1.0 μs per ligand. Restraints were gradually eliminated during the first 100 ns in order to avoid changes in the ligand binding mode due to structural fluctuations in the ATP-binding pocket, and the analysis of the trajectories was performed on the snapshots sampled in the last 150 ns unrestrained MD simulation.

### Human endothelial cell cultures and treatments

Cell culture studies were performed on a human endothelium cell line (EA.Hy926). Briefly, cells were seeded in 75 cm^2^ flasks with Dulbecco's modification of Eagle's High Glucose Medium (DMEM, Biowest), 10% fetal bovine serum (FBS, Biowest), penicillin (100 U/mL) and streptomycin (100 μg/mL) (Biowest) and kept at 37 °C in a humid atmosphere and 5% CO_2_. Once subconfluent (70–80%) subcultures were carried out using trypsin–EDTA (0.25%, Sigma) and used between passages 3 and 7. Cells were incubated for 1 h with either the AMPK activator AICAR (5 mM), IND6 (0.01, 0.1, 1, 10 and 100 µM); or 10 min with 2-DG (1 mM). There were also control cells (CT) without treatment. All the treatments were kept at 37 °C in a humid atmosphere and 5% CO_2_.

### Determination of the phospho-proteins by WB

The expression of the phosphorylated forms of: AMPKα in the residue Thr174 (pThr^174^-AMPKα; 62 kDa), ACC in the residue Ser79 (pSer^79^-ACC; 265 kDa), eNOS in the residue Ser 1177 (p-Ser^1177^eNOS; 133 kDa) and tubulin (55 kDa) were determined by WB^36^ (as described in Supplementary Material) in EA.Hy926 cells lysates. Cells were seeded in 6-well plates (Sarstedt) with a density of 120,000 cells/well. After 48 h, once subconfluent, they were treated with modulating compounds (AICAR at 5 mM, 2-deoxyglucose at 1 mM, and IND6 at 0.01, 0.1, 1, 10 and 100 μM) and in control untreated cells (CT). All the treatments were kept at 37 °C in a humid atmosphere and 5% CO_2_ for 1 h, except of the 2-deoxyglucose, which were maintained only during 10 min.

For the detection of all the proteins, acrylamide gels at 7% [H2O (5.1 mL); 1.5 M Tris–HCl pH = 8.8 (2.5 mL); SDS at 20% (50 μL) were used, acrylamide/bisacrylamide 30% (2.3 mL); ammonium persulphate 10% (50 μL), TEMED (5 μL)]. Polyclonal rabbit antibodies against the pSer^79^-ACC, pThr^174^-AMPK (1:500, Cell Signaling Technology) and pSer^1177^-eNOS (1:500, EMD Millipore Corporation) were used as primary antibodies and an anti-rabbit antibody (IgG) marked with peroxidase (1:2000, Santa Cruz Biotechnology) was used as secondary antibody. Tubulin was detected with a monoclonal mouse antibody (1:5000, Abcam) as primary antibody and an anti-mouse antibody (IgG) marked with peroxidase (1:10,000, GE Healthcare) as secondary. Quantification was carried out by establishing the relationship between the phosphorylated form of the different proteins and tubulin (p-protein/tubulin) based on the concentration of the modulating compounds administered, in addition to the negative control, on which no treatment was performed. It is worth to be mentioned that in order to obtain the maximum efficiency of the technique, WB gels were cut before the transference following the molecular weight markers depending on where the proteins were expected to appear.

### Detection of nitric oxide (NO) by fluorescence microscopy

EA. Hy926 cells were seeded on 8-well plates (Sarstedt) at a density of 6000 cells per well and allowed to grow in DMEM until a 60–70% confluence was reached. The medium was then aspirated and the cells for NO detection were incubated for 1 h with no treatment (control, CT), with DMSO 0.01% (maximum solvent concentration achieved with IND6 5 µM), AICAR 5 mM as a control of activation of NO production, IND6 1 µM and IND6 5 µM. Once the incubation time had elapsed, the medium was aspirated and the cells were incubated with 4.5-diaminofluorescein diacetate (Molecular Probes) (DAF-2DA 10^–5^ M) for 30 min in the dark, at 37 °C in a humid atmosphere and 5% CO_2_, in DMEM. The DAF-2DA is a non-fluorescent permeable probe capable of diffusing through the cell membrane. Inside the cell it is degraded by esterases to 4.5-diaminofluorescein-2 (2-DAF), which when reacting with intracellular NO gives rise to thiazolofluorescein (DAF-2T), capable of emitting green fluorescence (excitation wavelength 488 nm and emission wavelength 530 nm), so that the intensity of fluorescence emitted will be proportional to the production of NO in the cells. To assess eNOS involvement in DAF-2T fluorescence increase, endothelial cells were also incubated 30 min with the eNOS inhibitor L-NAME (100 µM) before stimulation with 1 and 5 µM IND6. The medium was then removed, the wells were washed twice with PBS for 15 min and then incubated with 4′,6-diamino-2-phenylindol (DAPI, 1 μg/mL; Molecular Probes) for 15 min at room temperature and in the dark. The DAPI (excitation wavelength 405 nm and emission wavelength 430 nm) stains the cell nuclei that are visualized by fluorescence microscopy. The cells were fixed in paraformaldehyde (PFA) for 1 h and washed 4 times with PBS 5 min. They were kept in PBS in darkness and at 4 °C until they were used for fluorescence microscopy (LeicaDM 2 000 Led). The quantification of the fluorescence intensity was performed with the ImageJ software and the fluorescence intensity at 530 nm is represented with respect to the control, which are the untreated cells in percentage form.

### Animals

Eight-week-old male Wistar rats were housed under controlled dark–light cycles (12 h:12 h from 8:00 to 20:00) and temperature (25 °C) conditions with standard food and water ad libitum. Animals were housed individually for two weeks. Then, they were anesthetized with ketamine (Rompun, Bayer; 0.8 mg/100 g) and xylazine (Imalgene, Merial; 0.4 mg/100 g) and sacrificed by exsanguination. Thoracic aorta was dissected and used for vascular function assays. The investigation conforms to the Guide for the Care and Use of Laboratory Animals published by the US National Institute of Health (NIH publication No. 85-23, revised in 2011) and was approved by the Ethics Committee of Universidad Complutense de Madrid (PROEX 206/18). All efforts were made to avoid animal suffering in accordance with the ARRIVE guidelines for reporting experiments involving animals.

### Vascular reactivity in the thoracic aorta artery

Vascular function studies were performed on the thoracic aorta, as previously described^5,37^. Once isolated, it was placed in a Petri dish containing Krebs Henseleit (KH) solution (115 mM NaCl; 4.6 mM KCl; 2.5 mM CaCl_2_-2H_2_O; 25 mM NaHCO_3_; 1.2 mM KH_2_PO_4_; 1.2 mM MgSO_4_-7H_2_O; 0.01 mM EDTA; 11.1 mM glucose) at 4 °C. With the aid of a binocular lens (Leica GZ4), the blood was carefully removed from inside the vessel and the connective tissue and perivascular adipose tissue were separated. The artery was then cut into segments of 2 mm in length. The experiments were carried out in intact arteries with endothelium.

Each of these segments was inserted between two horizontal rigid steel wires (300 μm in diameter) according to the method described previously^5,37^. Arterial segments were placed in an organ bath containing a KH solution at 37 °C continuously bubbled with a mixture of 95% O_2_ and 5% CO_2_, maintaining a physiological pH between 7.3 and 7.4 constantly. These were subjected to an initial tension of 1.5 g; which was periodically readjusted for 45 min until stabilization. Once the preparations were stabilized, their functional integrity was checked with KCl (75 mM). This value represented 100% of vascular contraction for each of the arterial segments. Next, concentration–response relaxation curves were performed after contraction to a single dose of NA (10^−7^ M), both with acetylcholine to test the integrity of endothelial function and with the different AMPK activators (AICAR 10^−5^ M–8 × 10^−3^ M; A-769662 10^−9^ M–10^−4^ M or IND6 10^−9^ M–10^−4^ M) to assess vasodilatation. To test the possible involvement of both AMPK and eNOS in the IND6-dependent vasodilation, arteries were preincubated for 30 min with the AMPK and eNOS inhibitors, SBI-0206965 (10^−4^ M) and L-NAME (10^−4^ M), respectively. After each curve, the preparations were washed 3 times with KH solution, and a 20-min rest period was left between each curve to ensure that the effects observed in each curve were not due to the agents used previously. The relaxation results were expressed as percentage relaxation with respect to the previous contraction obtained with NA (10^−7^ M). The analysis of the recordings obtained was performed with the aid of ACQ Knowledge 3.9 software (BioPac Systems INC).

### Preparation of drugs

AICAR (Toronto Chemical Research) was prepared at a concentration of 1.3 × 10^−1^ M in distilled water and kept at − 20 °C until use. 2-deoxyglucose (Sigma) was prepared at a concentration of 10^−1^ M in distilled water and used immediately. SBI-0206965 (Sigma-Aldrich), A-769662 (Tocris Bioscience) and the different modulator candidates were prepared in DMSO (Sigma). NA (Sigma Aldrich) was prepared in a saline-ascorbic solution (0.9% NaCl/0.01% ascorbic acid), Ach and L-NAME (Sigma Aldrich) were prepared in 0.9% NaCl solution. A stock solution (10^−2^ M) was prepared for each of them and stored at − 20 °C until use (maximum 3 months).

### Statistical analysis

The results obtained were expressed as the arithmetic mean ± the standard error of the arithmetic mean (S.M.E.). Comparisons of the results obtained between individual groups were made using Shapiro Wilk's analysis of variance followed by a one or two-way ANOVA and a Dunnett test (parametric) or a Kruskal–Wallis (non-parametric) to analyze differences between experimental groups with the untreated control. Significantly different groups were considered when p < 0.05. GraphPad Prism 9 software (San Diego) was used for statistical analysis. The free software ImageJ was used for image analysis.

## Supplementary Information

Supplementary Information.

## Abbreviations

2T-DAF: Thiazolofluorescein (2T-DAF)
2-DAF: 4.5-Diaminofluorescein-2
2-DG: 2-Deoxyglucose
ACC: Acetyl-CoA carboxylase
Ach: Acetylcholine
ADaM: Allosteric drug and metabolite binding site
ADP: Adenosine diphosphate
AICAR: 5-Aminoimidazole-4-carboxamide riboside
Akt: Protein kinase B
AMP: 5′-Adenosine monophosphate
AMPK: Protein kinase activated by 5′-adenosine monophosphate
ANOVA: Analysis of variance
ATP: 5′-Adenosine triphosphate
αAID: Self-inhibiting domain of the alpha subunit of AMPKα
αKD: Domain kinase of the alpha subunit of AMPKα
CBM: Carbohydrate binding site of AMPKβ
CBS: Cystathionine β-synthase
CM5: Carboxymethyldextran 5
DAPI: 4′,6-Diamino-2-phenylindol
DG-PO4: 2-Deoxyglucose phosphorylated by hexokinase
DMF: Dimethylformamide
DMSO: Dimethyl sulfoxide
EA. Hy926: Human endothelial cell line
EC_50_: Half-maximum effective concentration
EDC: 1-Ethyl-3-(3-dimethylpropanopropyl)-carbodiimide
E_max_: Pharmacological efficacy
eNOS: Endothelial nitric oxide synthase
Ki: Inhibition constant
$K^{\prime}_{i}$: Apparent inhibition constant
Ks: Dissociation constant
LC MS/MS: Proteomic technique that consists of performing a liquid chromatography followed by two consecutive mass spectrometry analyses
LKB1: Liver Kinase B1
L-NAME: *N*(ω)-Nitro-l-arginine methyl ester
MD: Molecular dynamics
mTOR: Mammalian/mechanistic target of rapamycin
NA: Noradrenaline
NHS: *N*-Hydroxysuccinimide
NO: Nitric oxide
p-ACC: Acetyl-CoA carboxylase phosphorylated in Ser79
p-AMPK: AMPK phosphorylated in Thr174
p-eNOS: Endothelial nitric oxide synthase phosphorylated in Ser1177
PFA: Paraformaldehyde
PI3K: Phosphatidyl inositol 3 kinase
RU: Resonance units
SAMS: Substrate for AMP-activated kinases
S.E.M.: Standard error of mean
S.D.: Standard deviation
SPR: Surface plasmon resonance
WB: Western blot or immune detection
