# Supplementary material for: Novel indolic AMPK modulators induce vasodilatation through activation of the AMPK–eNOS–NO pathway
Source: Sci Rep. 2022 Mar 10;12:4225. doi: 10.1038/s41598-022-07077-8 (PMC8913687; doi:10.1038/s41598-022-07077-8)

**Supplementary material**

**Novel indolic AMPK modulators induce vasodilatation through activation of the AMPK-eNOS-NO pathway**

**Marta Sanz-Gómez^1, 2#^, Elnaz Aledavood^3#^, Marina Beroiz^1^, Laura Lagartera^2^, Elena Vega-Martín^1^, Marta Gil-Ortega^4^, Jose Cumella^2^, Concepción Pérez^2^**, **Francisco Javier Luque**^3^**, Carolina Estarellas**^3*^**, María S Fernández-Alfonso^1*^, Ana Castro^2*^**

^1^Instituto Pluridisciplinar and Facultad de Farmacia, Universidad Complutense de Madrid, Spain

^2^Instituto de Química Médica, IQM-CSIC, Spain

^3^Departamento de Nutrición, Ciencias de la Alimentación y Gastronomía, Facultad de Farmacia y Ciencias de la Alimentación, Instituto de Biomedicina (IBUB) e Instituto de Química Teórica y Computacional (ICTQBUB), Universidad de Barcelona, Campus Torribera, Santa Coloma de Gramenet, Spain

^4^Departamento de Ciencias Farmacéuticas y de la Salud, Facultad de Farmacia, Universidad San Pablo-CEU, Madrid, Spain

**^#^** These authors contributed equally to this work.

*****Correspondence: acastro@iqm.csic.es (A.C.); marisolf@ucm.es (M.S.F-A.); cestarellas@ub.edu (C.E.)

**Table of contents**

**Synthesis of synthetic intermediates and final compounds**

- Synthesis of 3-Iodo-1H-indole-2-ethyl carboxylate (**2**)
- General synthesis for ethyl 3-aryl-1*H*-indole-2-carboxylates (**3-5** and **9**)
- Synthesis of ethyl 3-(4-(2-morpholinoethoxy)phenyl)-1H-indole-2-carboxylate (**10**)
- General procedure for the synthesis of the corresponding acids (**IND7, IND8, IND11**)

**Western Blot detailed complementary methods**

**Original unedited Western Blot gels**

All reagents were of commercial quality. Solvents were dried and purified by standard methods. Analytical TLC was performed on aluminum sheets coated with a 0.2 mm layer of silica gel 60 F254. Silica gel 60 (230−400 mesh) was used for flash chromatography. Analytical HPLC-MS was performed on Waters equipment coupled to a single quadrupole ESI-MS (Waters Micromass ZQ 2000) using a reverse-phase SunFire C18 4.6 × 50 mm column (3.5 μm) at a flow rate of 1 mL/min and by using a diode array UV detector. Mixtures of CH_3_CN and H_2_O were used as mobile phase (gradient of 15−95% of acetonitrile in water, as indicated in each case). HRMS (EI+) was carried out on Agilent 6520 Accurate-Mass Q-TOF LC/MS equipment. NMR spectra were recorded on a Bruker-AVANCE 300, a Varian-INOVA 400 and VARIAN SYSTEM-500 spectrometer. Melting points were determined on a Mettler MP70 apparatus and are uncorrected.

**3-Iodo-1H-indole-2-ethyl carboxylate (2)**

To a solution of NIS (2.25 g, 12 mmol) in DMF (20 mL) at 0°C, a solution of 1*H*-indole 2-ethyl carboxylate (1.89 g, 10 mmol) in DMF (15 mL) was slowly added. The mixture was stirred at room temperature for 1 hour. Then, a solution of sodium thiosulfate at 10% (5 mL) and water (10 mL) were added. The mixture was stirred at room temperature for another hour and the precipitate was collected by filtration to afford 2.9 g (96%) of **2** as a white solid. MS (ES, positive mode): 315 (M+H)^+^. ^1^H NMR (400 MHz, DMSO-d_6_) δ 12.25 (s, 1H), 7.78 – 6.88 (m, 4H), 4.38 (q, J = 7.1 Hz, 2H), 1.39 (t, J = 7.1 Hz, 3H).

**General synthesis for** **ethyl 3-aryl-1*H*-indole-2-carboxylates**

To a solution of ethyl 3-iodo-1*H*-indole-2-carboxylate (**2**) (1 eq.), tetrakis (triphenylphosphine) palladium (0) (0.05 eq.) and the corresponding boronic acid (1.2 eq.) in a solvent mixture of toluene:ethanol:water:1,4-dioxane 1:3:6:10 (40 mL), a solution of K_2_CO_3_ (4 eq.) in water (5 mmol/mL) was slowly added while stirring under argon. After 18 hours at 85°C, the mixture was concentrated in vacuo, and water (20 mL) was added. The mixture was extracted with AcOEt (3 x 20 mL). The organic phase was dried over anhydrous sodium sulfate, filtered and concentrated in vacuo. The crude was purified by chromatographic column using Hex/AcOEt (4:1) as eluent to afford, after concentration and high vacuum-drying, the corresponding aryl products.

**Ethyl 3-([1,1'-biphenyl]-4-yl)-1*H*-indole-2-carboxylate (3)**

Prepared from **2** (300 mg, 0.95 mmol), tetrakis (triphenylphosphine) palladium (0) (54 mg, 0.47 mmol), [1,1'-biphenyl]-4-ylboronic acid (226 mg, 1.10 mmol). Column chromatography on silica gel (Hex/AcOEt 4:1) by following the general procedure described for ethyl 3-aryl-1*H*-indole-2-carboxylates. Yield 170 mg (53 %). HPLC (SunFire): t_R_= 9.33 min (gradient: 15−95% of acetonitrile in water); MS (ES, positive mode): 342 (M + H)^+^. ^1^H NMR (400 MHz, CDCl_3_) δ 8.97 (s, 1H), 7.65 – 7.55 (m, 7H), 7.43 – 7.35 (m, 3H), 7.33 – 7.26 (m, 2H), 7.09 (ddd, *J* = 8.1, 6.9, 1.1 Hz, 1H), 4.25 (q, *J* = 7.1 Hz, 2H), 1.19 (t, *J* = 7.1 Hz, 3H).

**Ethyl 3-(4-(benzyloxy)phenyl)-1*H*-indole-2-carboxylate (4)**

Prepared from **2** (291 mg, 0.90 mmol), tetrakis (triphenylphosphine) palladium (0) (42 mg, 0.03 mmol) and 4-(benzyloxy)phenyl)boronic acid (250 mg, 1.10 mmol). Column chromatography on silica gel (Hex/AcOEt 4:1) by following the general procedure described for ethyl 3-aryl-1*H*-indole-2-carboxylates. Yield 315 mg (92 %). HPLC (SunFire): t_R_= 11.0 min (gradient: 15−95% of acetonitrile in water); MS (ES, positive mode): 372 (M + H)^+ 1^H NMR (300 MHz, CDCl_3_) δ 9.23 (s, 1H), 7.64 – 6.80 (m, 13H), 4.99 (s, 2H), 4.19 (q, *J* = 7.1 Hz, 2H), 1.11 (t, *J* = 7.1 Hz, 3H).

**Ethyl 3-(4-phenoxyphenyl)-1*H*-indole-2-carboxylate (5)**

Prepared from **2** (1.0 g, 3.17 mmol), tetrakis (triphenylphosphine) palladium (0) (179 mg, 0.15 mmol), (4-phenoxyphenyl)boronic acid (814 mg, 3.80 mmol). Column chromatography on silica gel (Hex/AcOEt 4:1) by following the general procedure described for ethyl 3-aryl-1*H*-indole-2-carboxylates. Yield 891 mg (78 %). HPLC (SunFire): t_R_= 9.30 min (gradient: 15−95% of acetonitrile in water). MS (ES, positive mode): 358 (M + H)^+^. ^1^H NMR (300 MHz, DMSO-*d*_6_) δ 11.90 (s, 1H), 9.34 (s, 1H), 7.55 – 7.49 (m, 4H), 7.47 – 7.39 (m, 2H), 7.35 – 7.28 (m, 1H), 7.21 – 7.05 (m, 4H), 6.91 – 6.74 (m, 1H), 4.24 (q, *J* = 7.1 Hz, 2H), 1.21 (t, *J* = 7.1 Hz, 3H).

**Ethyl 3-(4-hydroxyphenyl)-1*H*-indole-2-carboxylate (9)**

## Prepared from 2 (1.0 g, 3.10 mmol), tetrakis (triphenylphosphine) palladium (0) (179 mg, 0.15 mmol), 4-hydroxyphenylboronic acid (524 mg, 0.38 mmol). Column chromatography on silica gel (Hex/AcOEt 4:1) by following the general procedure described for ethyl 3-aryl-1*H*-indole-2-carboxylates. Yield 350 mg (40 %). HPLC (SunFire): t_R_= 7.83 min (gradient: 15−95% of acetonitrile in water). MS (ES, positive mode): 282 (M + H)^+^. ^1^H NMR (300 MHz, DMSO-d_6_) δ 11.75 (s, 1H), 9.46 (s, 1H), 7.52 – 7.45 (m, 2H), 7.34 – 7.25 (m, 3H), 7.10 – 7.04 (m, 1H), 6.87 – 6.81 (m, 1H), 4.23 (q, J = 7.1 Hz, 2H), 1.21 (t, J = 7.1 Hz, 3H).

**Synthesis of ethyl 3-(4-(2-morpholinoethoxy)phenyl)-1H-indole-2-carboxylate (10)**

To a solution of ethyl 3-(4-hydroxyphenyl)-1*H*-indole-2-carboxylate (**9**) (50 mg, 0.17 mmol.) in EtOH (10 mL) at 0°C, K_2_CO_3_ (73 mg, 0.51 mmol.) and 4-(3 chloropropyl)morpholine hydrochloride (42 mg, 0.23 mmol) were added. After 18 hours at reflux, water was added and the organic solvent was removed at reduced pressure. The organic phase was extracted with AcOEt (3 x 20 mL), and the organic extracts were washed with H_2_O. After drying over MgSO_4_, the solvent was removed to dryness and the resulting residue was purified by column chromatography, using AcOEt/Hex (1:4) as eluent, to afford 12 mg (18%) of the title compound. HPLC (SunFire): t_R_= 5.18 min (gradient: 15−95% of acetonitrile in water). MS (ES, positive mode): 394 (M + H)^+^. ^1^H NMR (300 MHz, CD_3_OD) δ 8.56 (s, 1H), 7.54 – 7.37 (m, 4H), 7.28 (ddd, *J* = 8.2, 6.9, 1.1 Hz, 1H), 7.11 – 6.93 (m, 3H), 4.32 – 4.14 (m, 4H), 3.77 – 3.69 (m, 4H), 2.83 (t, *J* = 5.5 Hz, 2H), 2.68 – 2.57 (m, 4H), 1.25 (t, *J* = 7.1 Hz, 3H).

**General procedure for the synthesis of the corresponding acids**

To a solution of the corresponding ester (1 eq) in EtOH (30 mL), KOH (4 eq.) dissolved in H_2_O (5 mmol/mL) was added. After stirring at 100°C for 18 hours, HCl 1N (2 mL) was added. The resultant solid was filtrated and dried under reduced pressure to give the corresponding acids.

**3-([1,1'-Biphenyl]-4-yl)-1*H*-indole-2-carboxylic acid (IND7)**

Prepared from **3** (100 mg, 0.30 mmol) and KOH (70 mg, 1.20 mmol) by following the general procedure described for 3-aryl-1*H*-indole-2-carboxylic acid. Yield 18 mg (18%). HPLC (SunFire): t_R_= 9.71 min (gradient: 15−95% of acetonitrile in water). MS (ES, positive mode): 314 (M + H)^+^ Mp 219ºC-222ºC. ^1^H NMR (300 MHz, DMSO-d_6_) δ 11.24 (s, 1H), 7.80 – 7.26 (m, 11H), 7.11 (ddd, *J* = 8.1, 6.9, 1.2 Hz, 1H), 6.98 (ddd, *J* = 8.1, 6.9, 1.1 Hz, 1H). ^13^C NMR (75 MHz, DMSO-d_6_) δ 140.8, 137.4, 135.4, 134.9, 131.5, 129.3, 127.9, 127.4, 126.79, 125.9, 122.8, 119.9, 119.6, 112.6. HRMS (EI+) m/z ([M]^+^) calcd for C_21_H_15_NO_3_ 313.11053; found 313.11028.

**3-(4-Phenoxyphenyl)-1*H*-indole-2-carboxylic acid (IND8)**

Prepared from **7** (100 mg, 0.28 mmol) and KOH (62 mg, 1.10 mmol) by following the general procedure described for 3-aryl-1*H*-indole-2-carboxylic acid. Yield 30 mg (32%). HPLC (SunFire): t_R_= 9.69 min (gradient: 15−95% of acetonitrile in water). MS (ES, positive mode): 330 (M + H)^+^. Mp 191ºC-193ºC. ^1^H NMR (300 MHz, DMSO-*d*_6_) δ 12.87 (s, 1H), 11.80 (s, 1H), 7.59 – 6.91 (m, 13H). ^13^C NMR (75 MHz, DMSO-*d*_6_) δ 161.7, 157.08, 156.0, 136.5, 132.4, 130.4, 129.1, 127.1, 125.4, 123.8, 123.0, 122.2, 120.9, 120.8, 119.0, 118.3, 113.0, 60.6, 14.3. HRMS (EI+) m/z ([M]^+^) calcd for C_21_H_15_NO_3_ 329.10656 found 329.10619

**3-(4-(2-Morpholinoethoxy)phenyl)-1*H*-indole-2-carboxylic acid (IND 11)**

Prepared from **10** (120 mg, 0.30 mmol) and KOH (67 mg, 1.20 mmol) by following the general procedure described for 3-aryl-1*H*-indole-2-carboxylic acid. Yield 32 mg (29%). HPLC (SunFire): t_R_= 4.46 min (gradient: 15−95% of acetonitrile in water). MS (ES, positive mode): 367 (M + H)^+^. Mp 206ºC-208 ºC. ^1^H NMR (500 MHz, DMSO-*d*_6_) δ 11.75 (s, 1H), 7.54 – 7.38 (m, 4H), 7.26 (ddd, *J* = 8.2, 6.9, 1.2 Hz, 1H), 7.11 – 6.92 (m, 3H), 4.52 (s, 2H), 4.03 – 3.81 (m, 4H), 3.65 – 3.45 (m, 4H), 3.23 (s, 3H). ^13^C NMR (125 MHz, DMSO-*d*_6_) δ 163.3, 136.4, 132.0, 127.5, 125.1, 123.8, 122.0, 120.9, 120.6, 114.5, 113.0, 63.6, 52.1. HRMS (EI+) m/z ([M]^+^) calcd for C_21_H_22_N_2_O_4_ 366.15957; found 366.15976.

**Western blot analysis**

Adapted from Hawley *et al.* [39]. Western Blot (WB) studies were conducted in treated and CT EA. Hy926 cells dissolved in ice cold lysis buffer (50mM Na4P2O7, 50mM NaF, 5mM EDTA, 5mM NaCl, 5mM EGTA, 10mM HEPES, 0.5% Triton, 1µg/mL leupeptin, 1µg/mL aprotinin, 0.5µL/mL N-α-p- tosyl-l-lysine in chloromethyl ketone and 20µM phenylmethylsulfonyl fluoride) and frozen immediately at -80ºC until use. Just before gel running, they were centrifuged at 13,000 rpm at 4°C for 20min (MiniSpin Plus, Eppendorf) and the supernatant was collected. Protein concentration was determined by colorimetry using Protein Assay (Bio-Rad) in a 96-well plate. After an incubation of 5min at 25°C, absorbance was determined at a wavelength of 595nm. A standard albumin curve (SAB) was used to quantify the protein concentration. Protein concentration was adjusted to 1 mg/mL protein with Laemli [50mM Tris, (pH=6.8), 10% glycerol, 10% sodium dodecyl sulfate (SDS), 5% β-mercaptoethanol, 2mg/mL bromophenol blue] and separated by electrophoresis, using a MiniProtean3 (Bio-Rad) system. The samples were boiled in a bath at 100°C for 5min and loaded (17µg protein/well) into a 7% acrylamide gel. PrecisionPlusProteinTM All blue standard (Bio-Rad) was used as a molecular weight marker. Electrophoresis was performed under denaturing conditions at 150V for 70min at 25ºC and in an electrode buffer (0.2 M glycine; 0.025M Tris and 0.1% SDS). Then, the gel proteins were transferred to a nitrocellulose membrane with a pore size of 0.2µm (Bio-Rad) through a transfer system at 25V for 10min for high molecular weight proteins (ACC) and for 7min for medium-high molecular weight proteins (<250KDa), such as eNOS, AMPK or tubulin (Trans-Blot Turbo Transfer, Bio-Rad). The membranes were washed twice for 5min in agitation with a washing solution [0.1% skimmed milk powder in phosphate buffer saline with 0.5% Tween 20 (PBS-T)]. In order to avoid non-specific binding, the membranes were incubated for 1h in agitation with a blocking solution (5% skimmed milk powder in PBS-T). Subsequently, they were washed 3 times for 5min and incubated at 4°C for 24h with the corresponding primary antibody at the concentration of 1:500 for p-ACC, p-eNOS and p-AMPK and 1:5,000 for tubulin. Primary antibodies were prepared in a specific solution (0.5% BSA; PBS-T and 0.05% azide). The following day, the membranes were washed 3 times for 5min and incubated for 1h at 25°C in agitation with the corresponding secondary antibody marked with peroxidase at the concentration of 1:4,000 for p-ACC and p-eNOS; 1:6,000 for p-AMPK and 1:10,000 for tubulin. This was prepared in a solution of 1% skimmed milk powder in PBS-T. Then, the membranes were washed again 3 times for 5min to remove the remains of the secondary antibody. The detection of the different proteins was carried out by chemiluminescence, using a detection kit (Enhanced Chemiluminescence ECL Prime, Amersham Bioscience) and subsequent exposure in a dark room for 1-2 minutes on autoradiographic film (GE Healthcare) for subsequent development with developer and fixer (Sigma). The quantification of the bands obtained by densitometry was carried out using ImageJ (Java Community Process) software of the scanner-digitalized films. Once the final image was merged, same contrast and bright corrections were performed in all the films in order to improve their interpretation.

**Original unedited Western Blot gels**

The original gel used for the edited Figure 3, after its trans-light scanning, cropping and contrast and bright adjustment, is marked within a red box.

**p-Ser79-ACC**


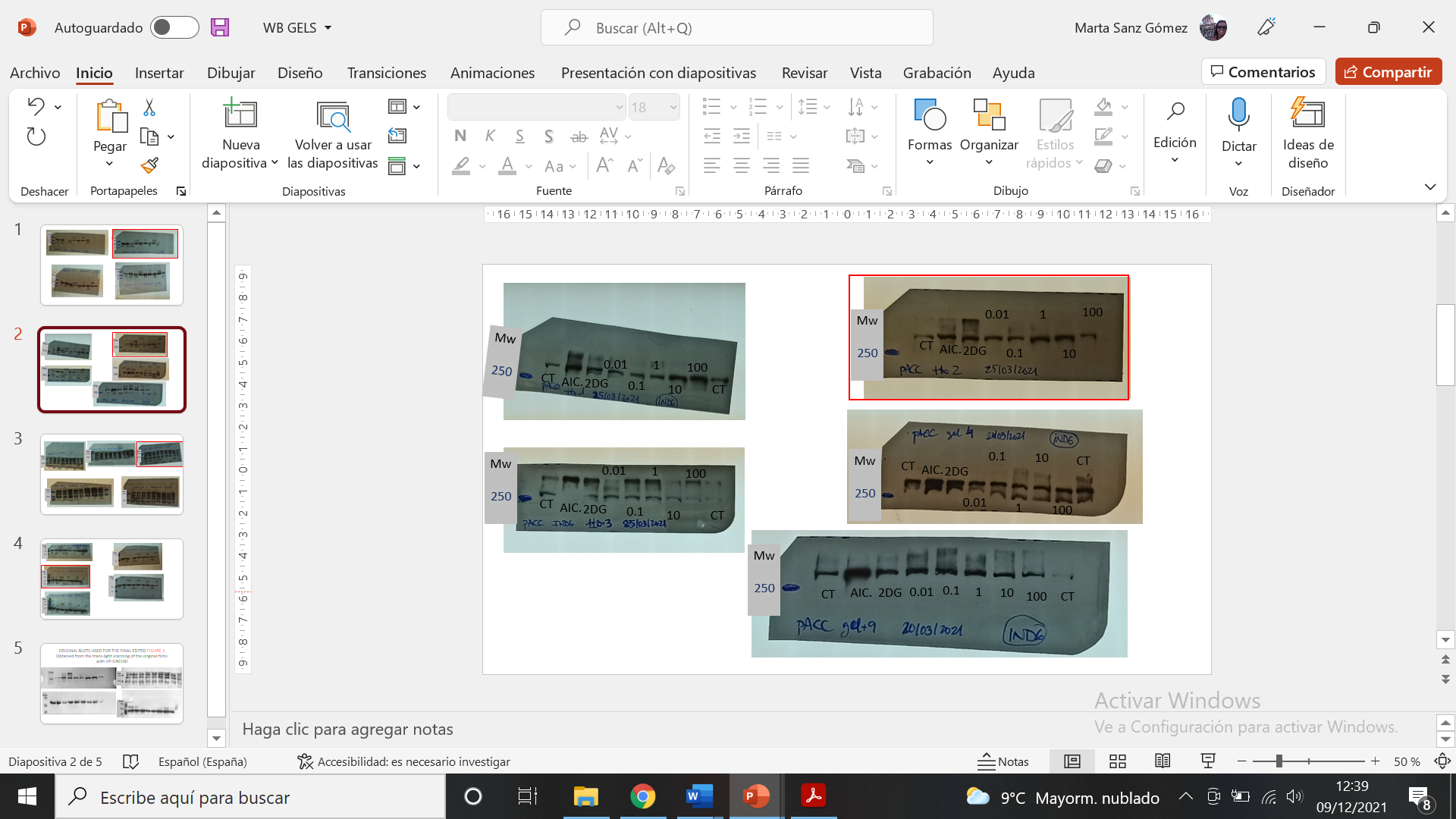


**p-Ser1177-eNOS**


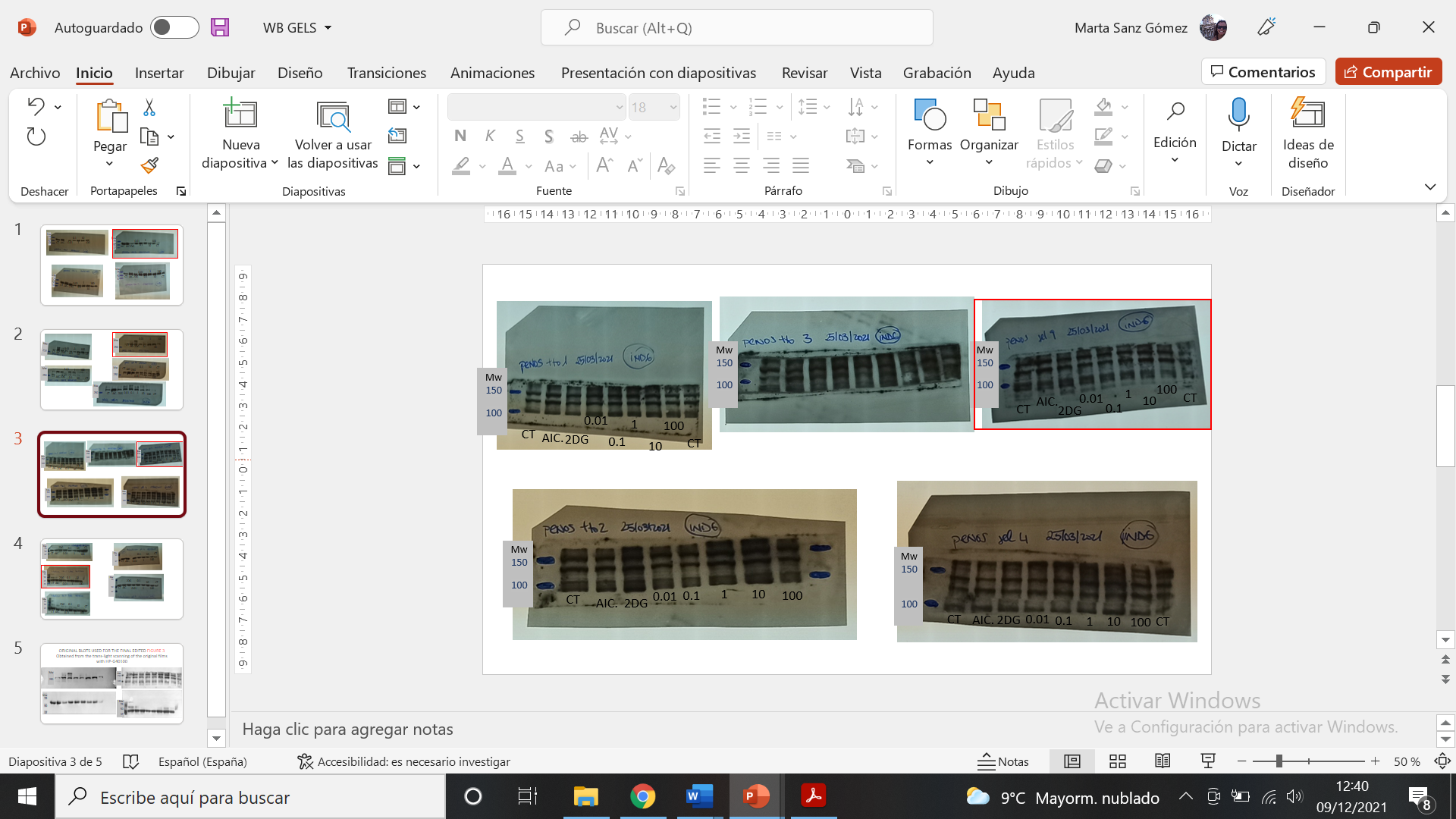


**p-Thr174-αAMPK**


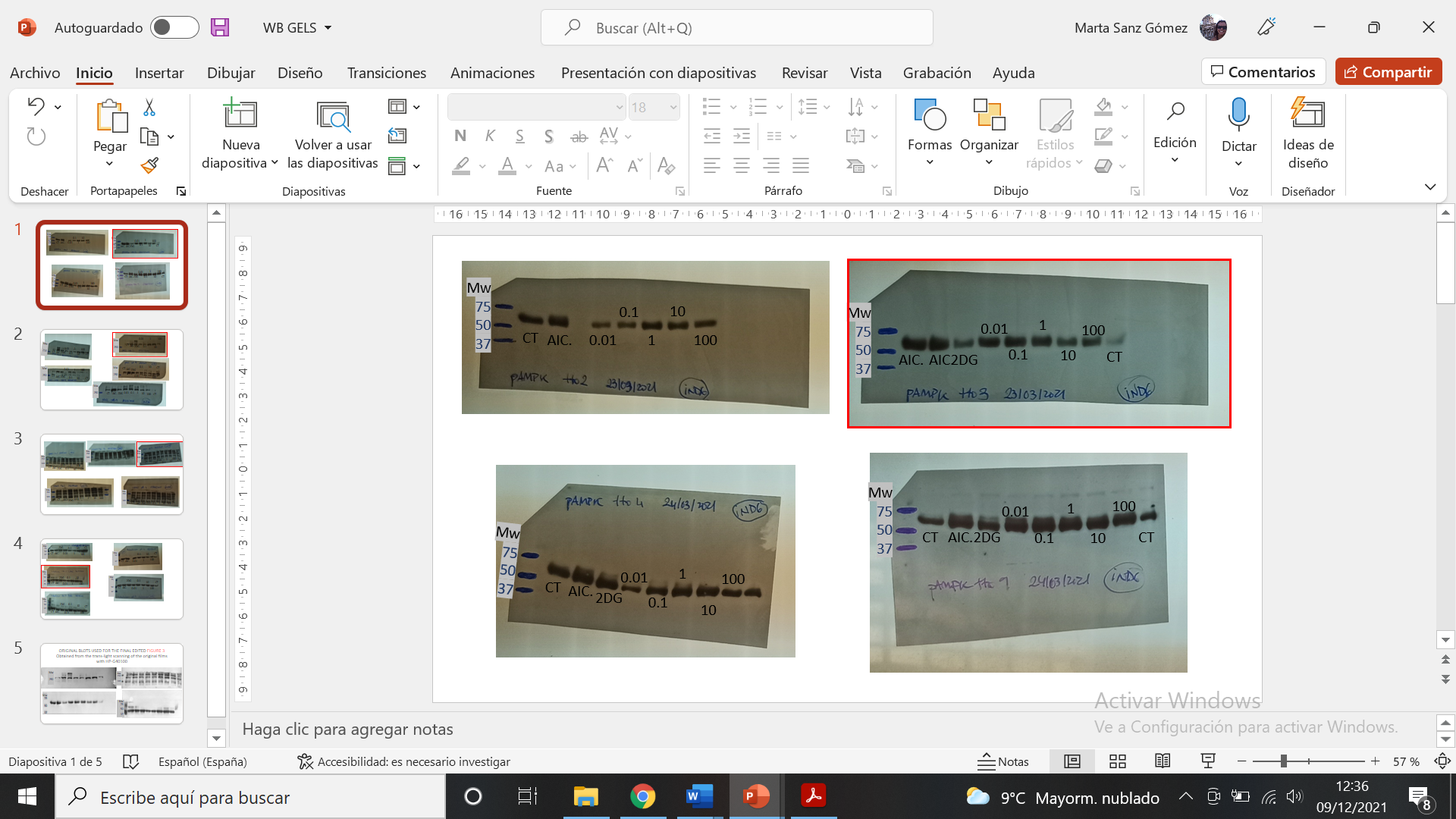


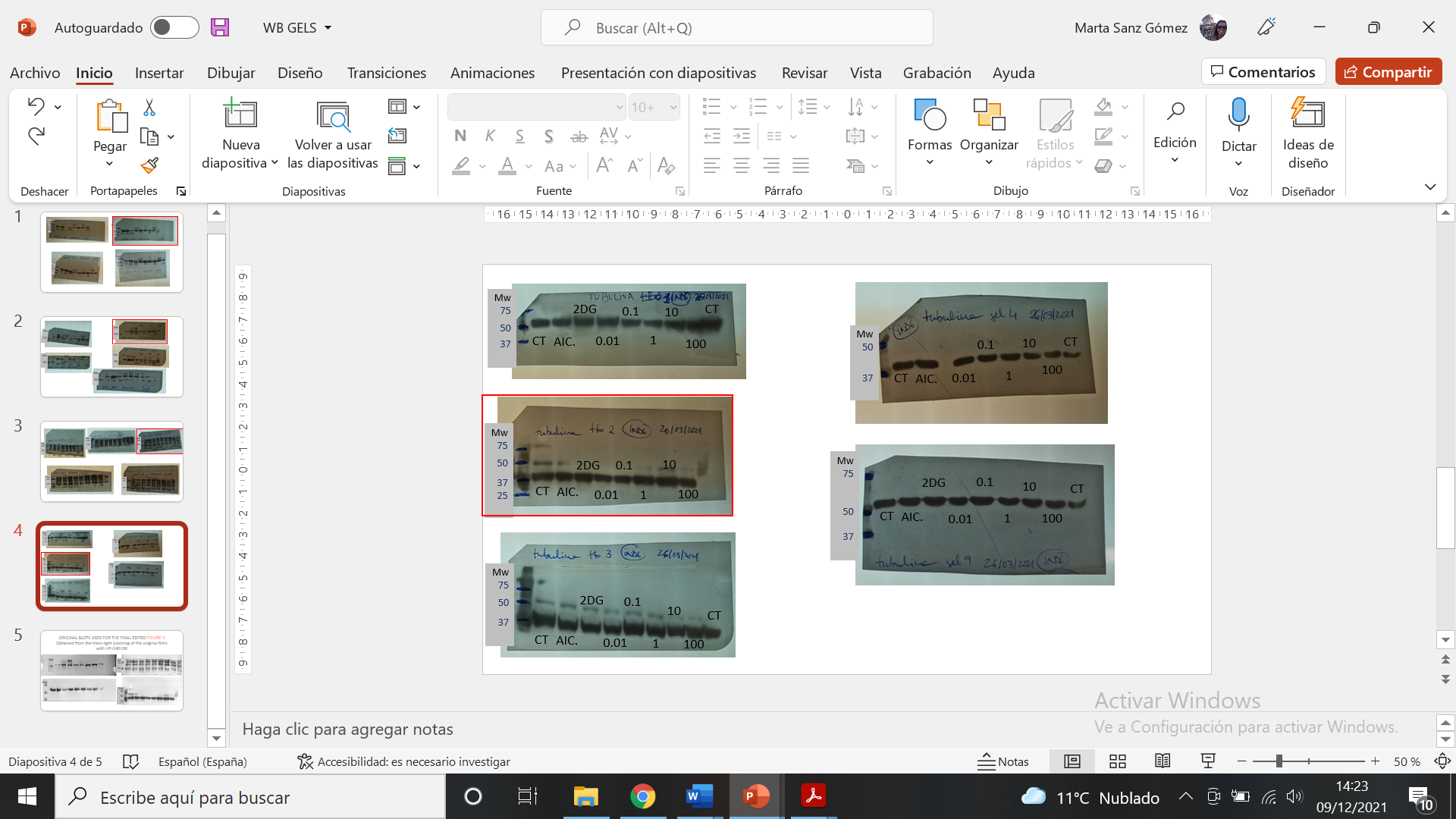
**Tubulin**

**Scanned blots used for Figure 3 composite:**

Obtained from the trans-light scanning of the original films with HP-G40100.

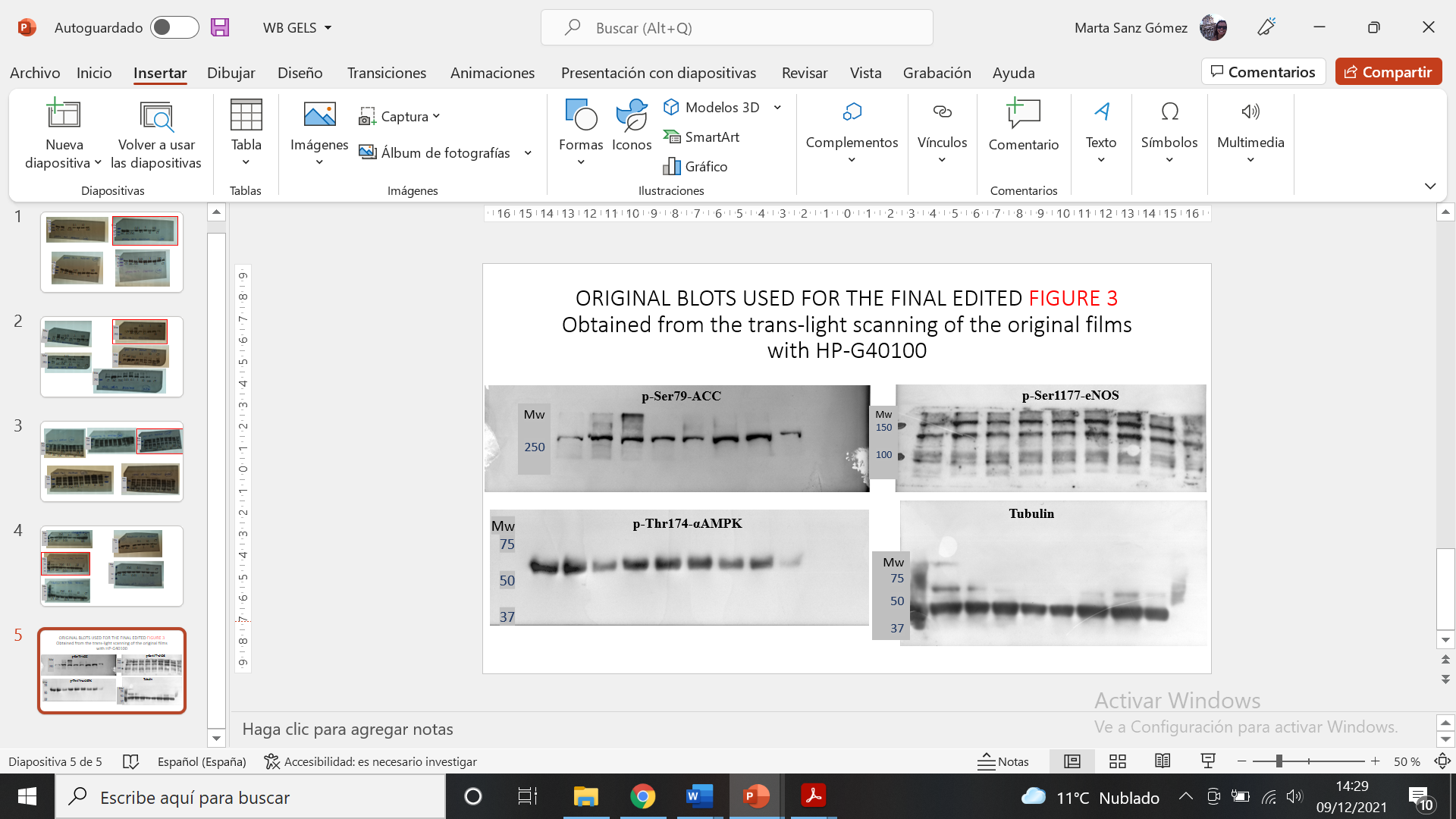


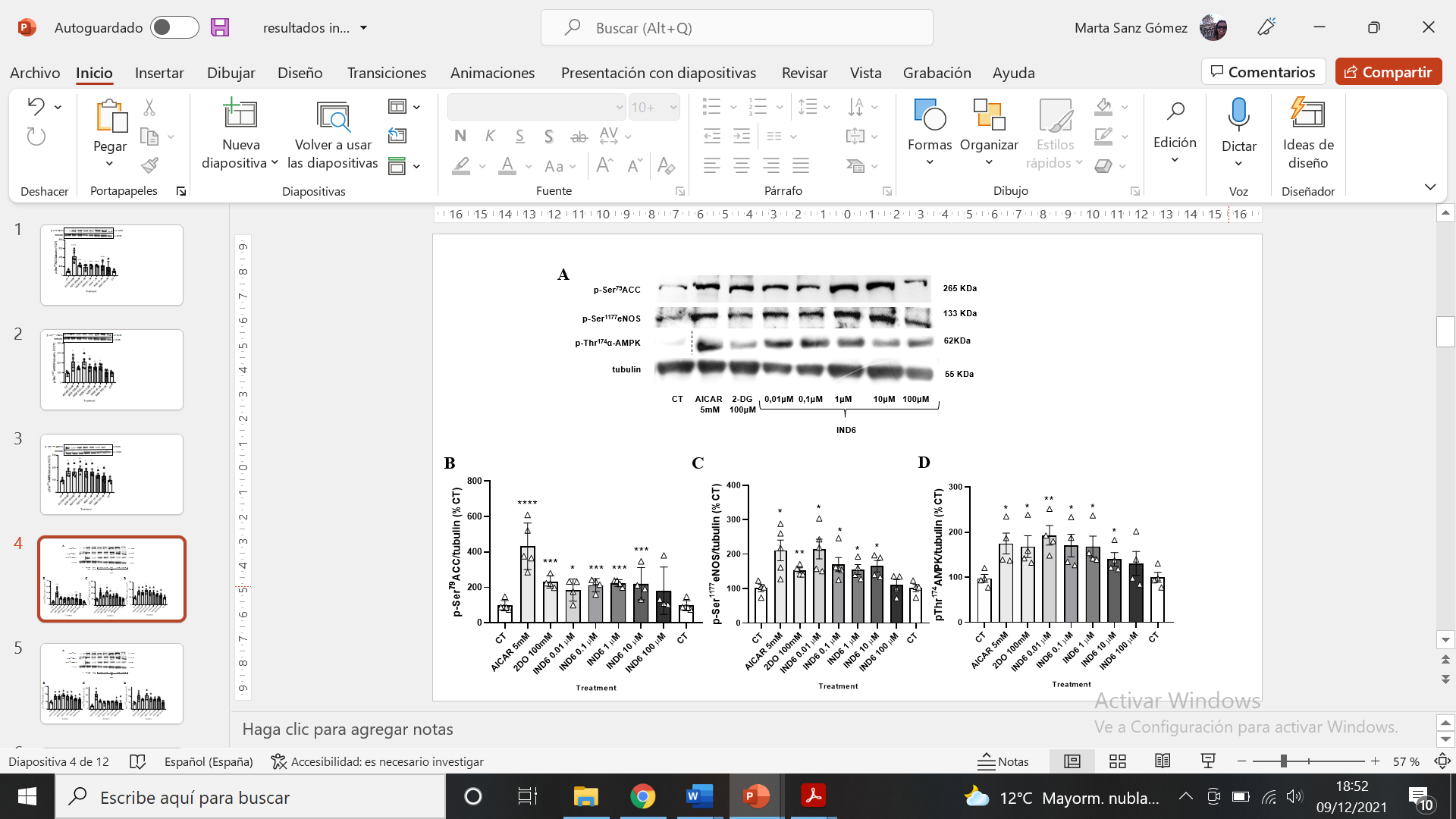

Supplement: Supplementary file 1 — Supplementary Information. [file 41598_2022_7077_MOESM1_ESM.docx]
